# Azacitidine combined with the selective FLT3 kinase inhibitor crenolanib disrupts stromal protection and inhibits expansion of residual leukemia-initiating cells in *FLT3*-ITD AML with concurrent epigenetic mutations

**DOI:** 10.18632/oncotarget.21877

**Published:** 2017-10-16

**Authors:** Anne-Kathrin Garz, Saskia Wolf, Sonja Grath, Verena Gaidzik, Stefan Habringer, Binje Vick, Martina Rudelius, Christoph Ziegenhain, Sylvia Herold, Marie-Theresa Weickert, Martha Smets, Christian Peschel, Robert A.J. Oostendorp, Sebastian Bultmann, Irmela Jeremias, Christian Thiede, Konstanze Döhner, Ulrich Keller, Katharina S. Götze

**Affiliations:** ^1^ Department of Medicine III, Klinikum rechts der Isar, Technische Universität München (TUM), Munich, Germany; ^2^ German Cancer Consortium (DKTK) and German Cancer Research Center (DKFZ), Heidelberg, Germany; ^3^ Department of Biology II, Ludwig-Maximilians-University (LMU), Munich, Germany; ^4^ Department of Internal Medicine III, University of Ulm, Ulm, Germany; ^5^ Helmholtz Zentrum München, German Research Center for Environmental Health, Munich, Germany; ^6^ Department of Pathology, Heinrich-Heine University Düsseldorf, Düsseldorf, Germany; ^7^ Department of Internal Medicine I, Gustav Carus University Dresden, Dresden, Germany

**Keywords:** FLT3-ITD, crenolanib, azacitidine, leukemia-initiating cell (LIC), TET2

## Abstract

Effectively targeting leukemia-initiating cells (LIC) in *FLT3*-ITD-mutated acute myeloid leukemia (AML) is crucial for cure. Tyrosine kinase inhibitors (TKI) have limited impact as single agents, failing to eradicate LIC in the bone marrow. Using primary AML samples and a patient-derived xenograft model, we investigated whether combining the FLT3-selective TKI crenolanib with the hypomethylating agent azacitidine (AZA) eliminates *FLT3*-ITD LIC and whether efficacy of this combination depends on co-existing mutations. Using multiparameter flow cytometry, we show *FLT3*-ITD occurs within the most primitive Lin^-^/CD33^(+)^/CD45^dim^/CD34^+^CD38^-^ LIC compartment. Crenolanib alone could not target *FLT3*-ITD LIC in contact with niche cells while addition of AZA overcame stromal protection resulting in dramatically reduced clonogenic capacity of LIC *in vitro* and severely impaired engraftment in NSG mice. Strikingly, *FLT3*-mutated samples harboring *TET2* mutations were completely resistant to crenolanib whereas neither *NPM1* nor *DNMT3A* mutations influenced response. Conversely, primary AML LIC harboring either *TET2, DNMT3A* or *NPM1* mutations did not show increased sensitivity to AZA.

In summary, resistance of *FLT3*-ITD LIC to TKI depends on co-existing epigenetic mutations. However, AZA + crenolanib effectively abrogates stromal protection and inhibits survival of *FLT3*-ITD LIC irrespective of mutations, providing evidence for this combination as a means to suppress residual LIC.

## INTRODUCTION

Acute myeloid leukemia (AML) with internal tandem duplications in the FLT3 tyrosine kinase (*FLT3*-ITD) remains a disease with dismal prognosis. Despite intensive chemotherapy and allogeneic stem cell transplantation as post-remission therapy, relapses are frequent. First, second and third generation tyrosine kinase inhibitors (TKI) have been evaluated for *FLT3*-mutated AML with varying degrees of success and only transient responses as single agents [1–4].

Reasons for the limited success of TKI in *FLT3*-mutated AML are diverse. Prolonged treatment with TKI leads to emergence of secondary kinase resistance mutations [5, 6]. Autocrine feedback loops in AML cells induce expression of FLT3 ligand (FL) [1]. Importantly, prevention of relapse requires complete eradication of the leukemic clone, including those rare leukemia-initiating cells (LIC) enriched in the CD34^+^CD38^-^ bone marrow (BM) compartment and responsible for disease propagation [7, 8]. LIC are protected from conventional chemotherapy by their BM microenvironment and we have previously shown that CD34^+^ LIC in *FLT3*-ITD AML persist and expand during treatment with first- and second generation TKI owing to their selective protection by stromal cells [9].

Developing more effective TKI and combining TKI with other novel agents such as epigenetic drugs are strategies currently being pursued in *FLT3*-ITD AML [10–12]. Several lines of evidence led us to hypothesize that combining a more selective FLT3 inhibitor with the hypomethylating agent azacitidine (AZA) may override the protective effect of the stromal microenvironment and thus more effectively eliminate LIC.

As a more potent FLT3 inhibitor, we chose crenolanib (creno), a promising new third-generation type I FLT3 inhibitor with high selectivity towards FLT3 [13, 14]. In contrast to type II TKI, it inhibits FLT3-ITD as well as TKD mutations by targeting both inactive and active conformational states and thus displays activity against kinase resistance mutations [13, 14]. *In vitro* studies have found creno to have superior cytotoxic effects compared to sorafenib (sora) or quizartinib and the compound is currently being evaluated in phase II trials in *FLT3*-ITD AML [14, 15]. Azacitidine (AZA) has emerged as a promising drug for the treatment of elderly AML and is also being evaluated as an add-on to intensive induction chemotherapy [16, 17]. Pre-emptive treatment with AZA can delay or prevent hematological relapses in AML with minimal residual disease, indicating it may have activity towards LIC [18]. In contrast to intensive chemotherapy, treatment with azacitidine does not appear to induce increased secretion of FL from the bone marrow [10, 19]. Furthermore, resistance of *FLT3*-mutated LIC to TKI may be mediated by co-mutations, the most frequent being either *NPM1* or epigenetic mutations (i.e. *DNMT3A*, *TET2 and IDH1/2*) [20, 21], which have have been postulated to show particular susceptibility to hypomethylating agents such as AZA [22–25]. Lastly, compensatory pathways upregulated in leukemia cells in response to TKI include survival pathways known to be mediated by stromal contact and which may be affected by AZA [26–29].

Here, using primary AML samples *in vitro* as well as in an *in vivo* xenograft model, we investigated whether creno in combination with AZA can eliminate LIC protected by stromal contact in *FLT3*-mutated AML and determined whether treatment response is dependent on co-existing genetic mutations in *DNMT3A*, *NPM1* and *TET2.*

## RESULTS

### *FLT3*-ITD is present within the CD34^+^CD38^-^ LIC-enriched compartment concurrent with *NPM1*, *DNMT3A* and *TET2* mutations

**Figure 1:**
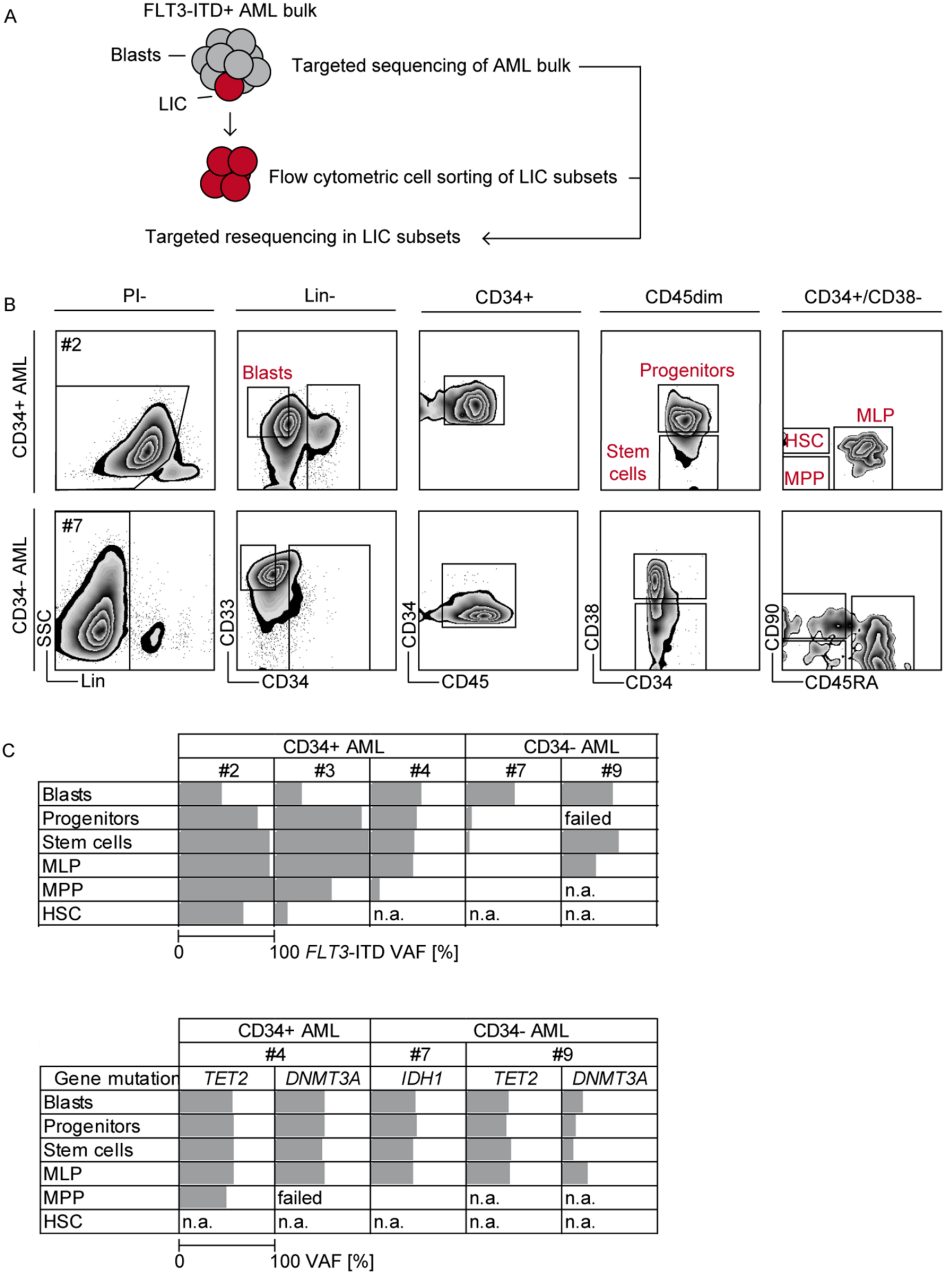
Detection of *FLT3*-ITD and concurrent gene mutations in leukemic stem/progenitor compartments Experimental design **(A)**. Gating strategy for multiparameter flow cytometric sorting of CD34^+^ and CD34^-^ AML BM samples. Blasts (Lin^-^/CD33^+^/CD34^-^), committed progenitors (Lin^-^/CD33^(+)^/CD45^dim^/CD34^+^CD38^+^), and early stem cell compartments (Lin^-^ /CD33^(+)^/CD45^dim^/CD34^+^CD38^-^) i.e. MLP (CD45RA^+^), MPP (CD45RA^-^CD90^-^) and HSC (CD45RA^-^CD90^+^). Representative plots for a CD34^+^ AML (upper panel, Table 1 patient ^#^2) and CD34^-^ AML sample (lower panel, Table 1 patient ^#^7) are shown **(B)**. gDNA was isolated from sorted compartments and mutations in *FLT3*, *NPM1*, *DNMT3A*, *TET2* and *IDH1/2* genes were detected by targeted resequencing. Variant allele frequencies (VAF, %) of *FLT3*-ITD (**C**, upper panel) and epigenetic mutations (C, lower panel) in sorted populations are shown. n.a., not available (e.g. population not found).

The prerequisite for durable responses in AML therapy is the successful elimination of LIC in the BM. It is well established by next-generation sequencing (NGS) data that mutations in epigenetic regulators such as *DNMT3A*, *TET2* and *IDH1/2* occur at a very early pre-leukemic stage within the primitive hematopoietic stem/progenitor cell compartment. In contrast, *FLT3*-ITD is considered a late event in leukemogenesis, but may often be the crucial initiator for leukemic expansion [30]. Only limited data exist suggesting presence of *FLT3*-ITD in LIC and sophisticated sorting was not performed in these studies, nor were results correlated to epigenetic mutations (unknown at the time) [31, 32]. Thus, to determine occurrence of *FLT3*-ITD in distinct phenotypically defined leukemic stem/progenitor subpopulations in relation to pre-leukemic and early leukemic hits, we analyzed ten *FLT3*-ITD AML samples (five CD34^+^ and five CD34^-^, Table 1, patients #1-10) by amplicon based sequencing of 54 genes related to myeloid neoplasms followed by flow cytometric sorting and targeted resequencing of identified alterations within leukemic stem/progenitor subpopulations (Figure 1A, Supplementary Figure 1A and Supplementary Table 1).

**Table 1:** AML patient sample characteristics

ID	Age	Sex	Cytogenetics	*FLT3*-ITD/WT ratio	*NPM1*	*DNMT3A*	*TET2*	Other	CD34
1	58	m	46, XY, del13(q)	0.166	wt	wt	wt	*IDH2*, *RUNX1*, *SF3B1*, *STAG2*	Pos
2	81	m	46, XY	0.713	mut	wt	wt	neg	Pos
3	47	f	46, XX	0.62	mut	wt	wt	*RAD21*	Pos
4	35	f	46, XX	0.848	wt	mut	mut	*STAG2*, *GATA2*, (*MLL-PTD*)	Pos
5	63	f	46, XX, t(6;9)	0.23	wt	wt	wt	neg	Pos
6	68	m	46, XY	0.18	mut	wt	mut	neg	Neg
7	31	f	46, XX	0.63	mut	wt	wt	*CEBPA*, *IDH1*	Neg
8	69	m	49, XY, +Y, +21, +22	0.302	mut	wt	wt	*IDH1*	Neg
9	63	f	46, XX	0.559	mut	mut	mut	*WT1*	Neg
10	74	f	46, XX	0.98	mut	wt	mut	*NPM1*, *TET2*	Neg
11	36	m	46, XY	5.141	mut	mut	wt	n.a.	Pos
12	59	m	46, XY, i(14)(q10)	1.707	wt	wt	wt	n.a.	Pos
13	43	f	46, XX, add(19)(13.1)	0.582	wt	wt	wt	n.a.	Pos
14	41	f	46, XX, t(9;11)(p22;q23)	0.789	wt	wt	wt	n.a.	Pos
15	59	f	46, XX	0.799	mut	wt	mut	n.a.	Neg
16	66	m	46, XY	8.911	mut	mut	wt	n.a.	n.a.
17	64	m	n.a.	0.625	mut	mut	wt	n.a.	n.a.
18	70	m	n.a.	0.095	wt	wt	mut	n.a.	n.a.
19	51	m	n.a.	0.861	wt	wt	wt	n.a.	n.a.
20	46	f	46, XX	1.687	mut	mut	wt	n.a.	Neg
21	63	f	46, XX, i(14)(q10) 46, idem, t(X;3)(p11;p13)	0.854	wt	mut	wt	n.a.	Neg
22	78	f	46, XX	0.552	mut	wt	mut	n.a.	Neg
23	72	f	46, XX	0.4	mut	n.a.	n.a.	n.a.	Pos
24	75	f	46, XX	0.8	wt	mut	wt	*JAK2*	Pos
25	59	f	46, XX	0.513	mut	mut	wt	n.a.	Neg
26	35	m	46, XY	0.836	mut	wt	wt	n.a.	Neg
27	61	f	46, XX	0.649	wt	wt	wt	RUNX1, NRAS	Pos
28	66	m	46, XY	0.151	mut	mut	wt	n.a.	Neg
29	77	f	46, XX	0.237	mut	wt	mut	n.a.	Neg
30	83	f	47,XX,+4	0.937	mut	mut	mut	n.a.	Pos
31	84	m	46,XY	0.885	mut	wt	mut	n.a.	Neg
32	76	m	46, XY, complex	1.294	wt	mut	mut	n.a.	Pos

M, male; f, female; mut, mutated; wt, wild-type; n.a., not available.

The mutational profile of our cohort was representative as it revealed a mean of 3.3 driver mutations per patient, concurrent with recent reports on the mutational landscape of AML [20, 21]. *NPM1* mutations were found in 7/10 patients. In 7/10 patients, epigenetic lesions in either *TET2*, *IDH1*, *IDH2* or *DNMT3A* were found. *IDH1/2* mutations appeared exclusive, whereas two patients (#4 and #9) with *DNMT3A* mutations also carried a *TET2* mutation. Only one patient (#5) had *FLT3*-ITD as the sole mutation, but showed an aberrant t(6;9) karyotype (Table 1, #5; Supplementary Figure 1A).

Next, we separated blasts, committed progenitors and early stem cell compartments by multiparameter flow cytometry based on the expression of CD33 as a known marker aberrantly expressed on LIC [33]. We found that the early leukemic stem cell compartment (Lin^-^/CD33^(+)^/CD45^dim^/CD34^+^CD38^-^) in all CD34^+^ AML samples (# 2, 3, 4, 5) was dominated by multilymphoid progenitors (MLP, CD45RA^+^), whereas in CD34^-^ AML, we also found multipotent progenitors (MPP, CD45RA^-^CD90^-^) and hematopoietic stem cells (HSC, CD45RA^-^CD90^+^, #7), consistent with published reports (Figure 1B and Supplementary Figure 1B) [34]. In all 5/10 AML samples where we were able to detect and sort leukemic HSC and MPP populations, *FLT3*-ITD was present in these fractions and variant allele frequencies (VAF) hierarchically increased from there (Figure 1C upper panel and Supplementary Table 1), indicating dominant clonal hematopoiesis. *NPM1* mutations were present in all analyzed compartments (Supplementary Table 1). *DNMT3A*, *TET2*, *IDH1/2* mutations were present in early and late leukemic stem/progenitor compartments with equally high VAF (Figure 1C lower panel and Supplementary Table 1), concurrent with the concept that these are founder mutations [35, 36].

In summary, we demonstrate that *FLT3*-ITD, *NPM1*, *DNMT3A* and *TET2* mutations frequently co-occur within the most primitive phenotypically definable Lin^-^/CD33^(+)^/CD45^dim^/CD34^+^CD38^-^ LIC compartments. We can therefore ascertain that *FLT3*-ITD is indeed an appropriate TKI target for LIC elimination in AML. However, co-occurrence of *NPM1*, *TET2* and *DNMT3A* mutations in this same compartment may influence response of *FLT3*-mutated LIC to TKI.

### *FLT3*-ITD cells are protected against crenolanib by stromal niche cells despite effective inhibition of *FLT3*-ITD signaling

**Figure 2:**
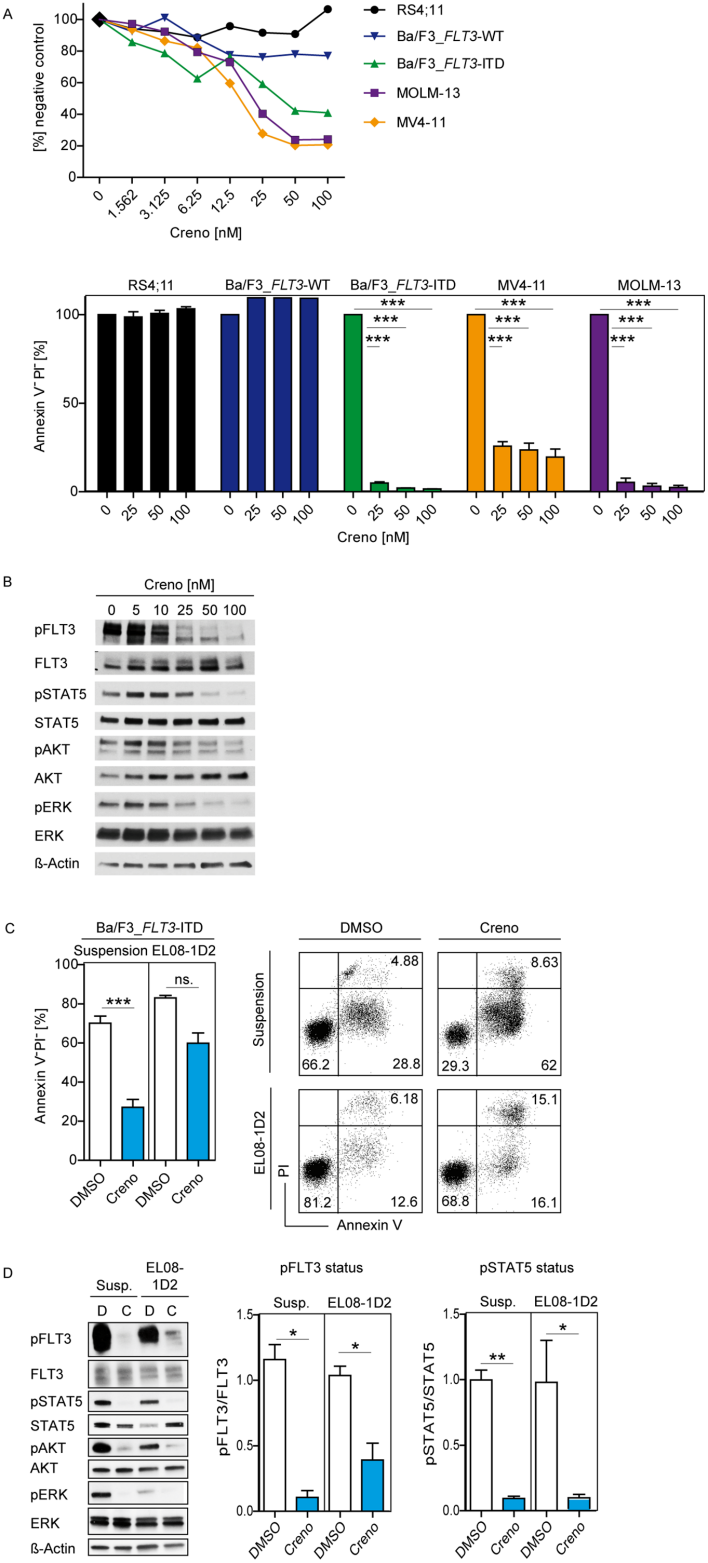
Efficacy of creno against *FLT3*-ITD leukemia cells is counteracted by stromal contact Dose response for leukemia cells treated with creno for 96 hours. Cell viability was determined by MTT assay. Results shown are mean ± SEM of triplicates (**A**, upper panel). Induction of cell death was measured by Annexin/PI staining of leukemia cells after treatment with creno (100 nM) for 96 hours. Percentage of live cells (defined as Annexin V^-^/PI^-^) was determined by flow cytometry. Results shown are mean ± SEM of triplicates (A, lower panel). Inhibition of FLT3-ITD signaling pathways assessed in Ba/F3_FLT3-ITD cells by immunoblotting after treatment with indicated creno concentrations for 1 hour. A representative blot is shown **(B)**. Ba/F3_*FLT3*-ITD cells were treated with DMSO or 100 nM creno in suspension or co-culture with EL08-1D2 for 24 hours. Results are shown as mean ± SEM of 3 independent experiments (**C**, left panel), and representative FACS plots (C, right panel). Representative immunoblot of *FLT3*-ITD signaling in Ba/F3_*FLT3*-ITD cells after treatment in mono- or co-culture (**D**, left panel). Intensity of FLT3- and STAT5-phosphorylation is shown as mean ± SEM of 3 independent experiments (D, right panel).

Before evaluating the efficacy of creno to target *FLT3*-ITD LIC, we confirmed the *FLT3*-ITD-selective effects of creno in leukemia cell lines. Creno was cytotoxic to murine Ba/F3 cells transduced with human *FLT3*-ITD (Ba/F3_*FLT3*-ITD) as well as *FLT3*-ITD human leukemia cell lines MV4-11 and MOLM-13 in a dose-dependent manner whereas no effect was observed on Ba/F3 cells transduced with *FLT3* wild-type receptor (Ba/F3_*FLT3*-WT) or the leukemia cell line RS4;11 (Figure 2A, upper panel). Creno-induced cell death was maximal at a concentration of 100 nM at 96 hours in *FLT3*-ITD cells (Figure 2A, lower panel) and FLT3 phosphorylation was effectively inhibited at 50 nM, as was phosphorylation of downstream targets STAT5, AKT and ERK (Figure 2B). Thus, we used a creno concentration of 100 nM for further experiments [14].

To test whether creno as single agent can induce cell death in *FLT3*-ITD driven Ba/F3 cells protected by stromal cells, we made use of an established *in vitro* co-culture system with the osteoblast-like murine embryonic cell line EL08-1D2 mimicking the BM niche and shown to effectively protect *FLT3*-ITD leukemia cells [9]. As previously observed by us and others, leukemia cells showed increased viability when cultured on stromal cells, highlighting their dependency on the BM microenvironment for survival [9, 37, 38]. Ba/F3_*FLT3*-ITD cells were sensitive to creno only when cultured in suspension, but almost completely protected from cytotoxic effects by culture on EL08-1D2 cells (Figure 2C), indicating that like first and second generation TKI, creno alone cannot effectively target *FLT3*-ITD cells in their niche. Linear regression analyses of this data showed a statistically significant interaction between stroma and creno, underscoring the conclusion that stroma confers resistance to creno in *FLT3*-mutated leukemia cells (complete statistical analyses in Supplementary data). Efficacy of creno to inhibit *FLT3*-ITD was not perturbed by stromal contact, as shown by persistent inhibition of pFLT3 and downstream targets pSTAT5, pAKT and pERK in the presence of stroma (Figure 2D and Supplementary Figure 2).

### Stromal resistance of *FLT3*-ITD AML cells to creno is overcome by addition of AZA

**Figure 3:**
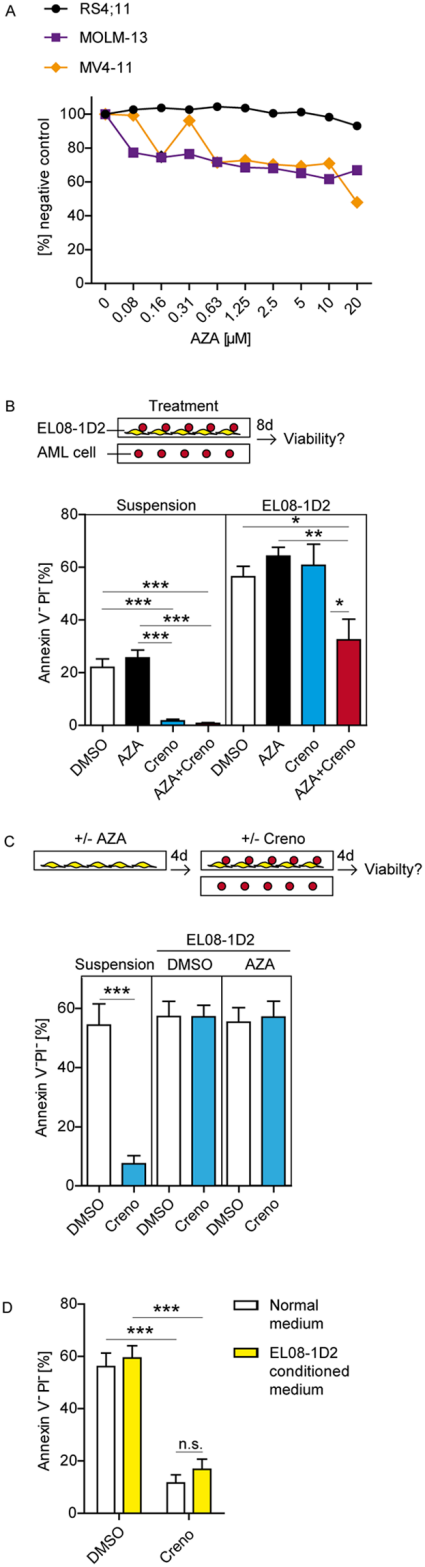
The addition of AZA to creno can overcome stromal resistance of *FLT3*-ITD leukemia cells Dose response for *FLT3*-ITD leukemia cells treated with AZA was determined by MTT after 96 hours. Results are shown as mean ± SEM of triplicates **(A)**. MV4-11 cells were cultured in suspension or on stromal EL08-1D2 layers. Cultures were treated on day 1 with either DMSO, 10 μM AZA, 100 nM creno or in combination. Induction of cell death was measured by Annexin V/PI staining after 8 days. Results are shown as mean ± SEM of three independent experiments **(B)**. EL08-1D2 cells were cultured in the presence or absence of 10 μM AZA for 4 days. The stromal cell layer was washed twice with PBS, MV4-11 cells were added and co-cultures were treated with DMSO or 100 nM creno for 4 days. AML cells were harvested and cell death was measured by Annexin V/PI staining, Results are shown as mean ± SEM of three independent experiments **(C)**. MV4-11 cells were cultured in standard medium or medium preconditioned by EL08-1D2 cells for 4 days. Cells were treated with 100 nM creno for 4 days and induction of cell death was measured as described above, Results are shown as mean ± SEM of three independent experiments **(D)**.

We then asked whether stromal resistance of *FLT3*-ITD cells to creno can be modulated by AZA. Azacitidine was added *in vitro* as a single dose on day 1 of the cell culture. Previously published analyses have shown that at 37°C azacitidine is stable in solution and induces distinct epigenetic changes in cells cultured for at least 72 hours after addition of the drug [39, 40]. Dose titration revealed that MV4-11 and MOLM-13 were only marginally sensitive to AZA up to 10 μM (Figure 3A), the dose corresponding to the maximum plasma concentration achieved in patients receiving AZA treatment [39, 41, 42]. Using this dose for further experiments, we assessed induction of apoptosis in *FLT3*-ITD homozygous MV4-11 cells cultured in suspension and on EL08-1D2 stromal layers in the presence of creno, AZA and the combination thereof (Figure 3B). Simultaneous combination of TKI and AZA induced most pronounced apoptosis of *FLT3*-ITD AML cells although stromal resistance was not overcome within 4 days of treatment (Supplementary Figure 3). As AZA incorporates into DNA as well as RNA, its anti-neoplastic effects are cell-cycle dependent and independent, respectively. Within four days, MV4-11 cells divide twice. Therefore, we asked whether combination therapy with AZA and TKI could be improved by prolonged drug exposure over 8 days (Figure 3B). The dependence of AML cells on stromal contact for survival was even more pronounced under prolonged culture conditions, as evidenced by reduced viability of untreated cells in suspension. Pro-longed Co-culture on stroma significantly increased viability of AML cells. AZA as a single agent did still not induce cell death. However, combined treatment with creno and AZA effectively reduced stromal resistance of *FLT3*-ITD AML cells indicated by significant induction of cell death in the presence of stroma (Figure 3B) and positive interaction testing by linear regression analyses (complete statistical analyses in Supplementary data). Nearly identical results were obtained with *FLT3*-ITD heterozygous MOLM-13 cells as well as using sora in combination with AZA (Supplementary Figures 3 and 4). Pre-treatment of EL08-1D2 cells with AZA did not sensitize MV4-11 cells to creno (Figure 3C) and stroma-derived soluble factors could not protect MV4-11 cells from creno (Figure 3D), implying that AZA perturbs stromal protection by effect on the direct interaction between *FLT3*-ITD cells and their niche.

### TKI response of *FLT3*-ITD LIC is dependent on concurrent genetic mutations

To translate our findings back to the situation in the patient, we then assayed LIC-enriched CD34^+^ cells from primary *FLT3*-ITD AML in our established *in vitro* BM model. A 4-day co-culture period was chosen for the primary samples as we have previously shown that CD34^+^ AML stem/progenitor cells divide at least once during this time period without losing expression of CD34 and prolonged *in vitro* culture of primary AML cells may lead to drug-independent loss of viability [9].

**Figure 4:**
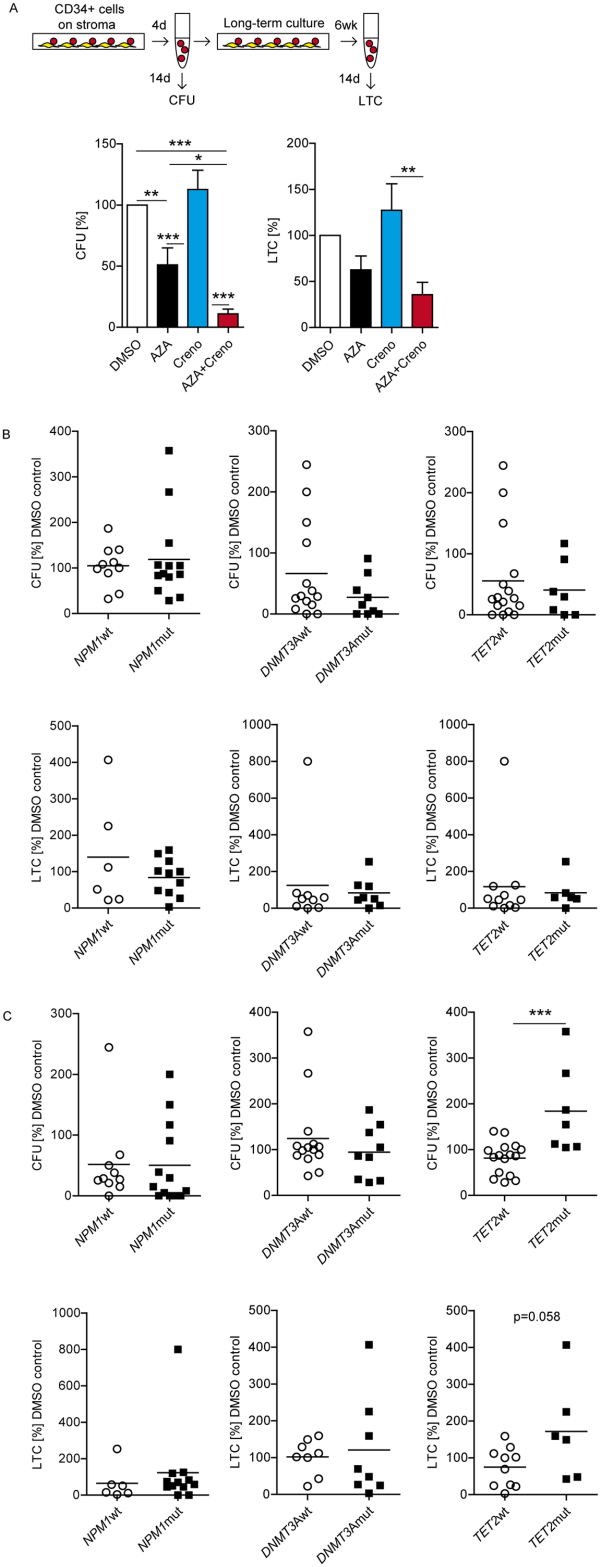
Sensitivity of primary *FLT3*-ITD LIC to crenolanib is dependent on concurrent epigenetic gene mutations Experimental design: enriched CD34^+^*FLT3*-ITD BM cells were cultured on EL08-1D2 stroma and treated on day 1 with DMSO, 10 μM AZA, 100 nM creno or the combination thereof. Cells were harvested after 4 days. Progenitor activity was assessed by short-term colony-forming unit assay (CFU) in methylcellulose. Long-term LIC capacity (long-term culture-derived colony-forming cells, LTC) was assessed after 6 weeks on irradiated (30 Gy) EL08-1D2 cells followed by plating in methylcellulose. Colonies were scored after 14 days using standard criteria. Results are shown as mean CFU (n = 23 ± SEM) and LTC frequencies (n = 17 ± SEM) in relation to DMSO controls **(A)**. Response to AZA alone regarding co-mutations in *NPM1* (CFU n = 23; LTC n =18), *DNMT3A* (CFU n = 23; LTC n =17), *TET2* (CFU n = 23, upper panels; LTC n =17, lower panels) **(B)**. Response to creno alone regarding co-mutations in *NPM1* (CFU n = 23; LTC n =18), *DNMT3A* (CFU n = 23; LTC n =16), *TET2* (CFU n = 23; LTC n =16) **(C)**.

*In vitro* treatment with creno, AZA or the combination was followed by colony forming cell assays (CFC) to determine short- as well as long-term proliferative potential characteristic of LIC (Figure 4A). We then correlated results with the presence of *DNMT3A*, *NPM1* and *TET2* mutations (Table 1). These are among the most common co-occurring mutations with *FLT3*-ITD and of particular interest in regard to epigenetic agents such as AZA [20, 25, 43].

In comparison to DMSO, AZA as a single agent reduced short-term and long-term colony growth of LIC by 49% and 37%, respectively (Figure 4A). As expected from our cell line data, creno alone did not prevent expansion of committed leukemic progenitors with short-term (CFU) or LIC with long-term proliferative potential (LTC) on stroma. Strikingly, the combination of AZA and creno eliminated 89% of short-term and 64% of long-term LIC despite stromal contact (Figure 4A). Of note, stromal protection of *FLT3*-ITD LIC towards inhibition by creno was not due to reactive FL secretion. Levels of stroma-derived FL were below detection threshold by ELISA regardless of treatment condition (data not shown) while levels of LIC-derived FL were unchanged (Supplementary Figure 5).

Contrary to expectation, response to AZA as a single agent was not influenced by presence of *DNMT3A*, *NPM1* or *TET2* mutations as determined by short-term and long-term proliferative potential (Figure 4B). Most strikingly, *TET2* mutations conferred resistance to creno alone, and *FLT3*-ITD/*TET2*mut cells expanded significantly during creno treatment, as evidenced by increased CFC numbers compared to DMSO in *FLT3*-ITD/*TET2*-mutated samples (Figure 4C). In contrast, neither *DNMT3A* nor *NPM1* mutations had an influence on response to creno (Figure 4C).

**Figure 5:**
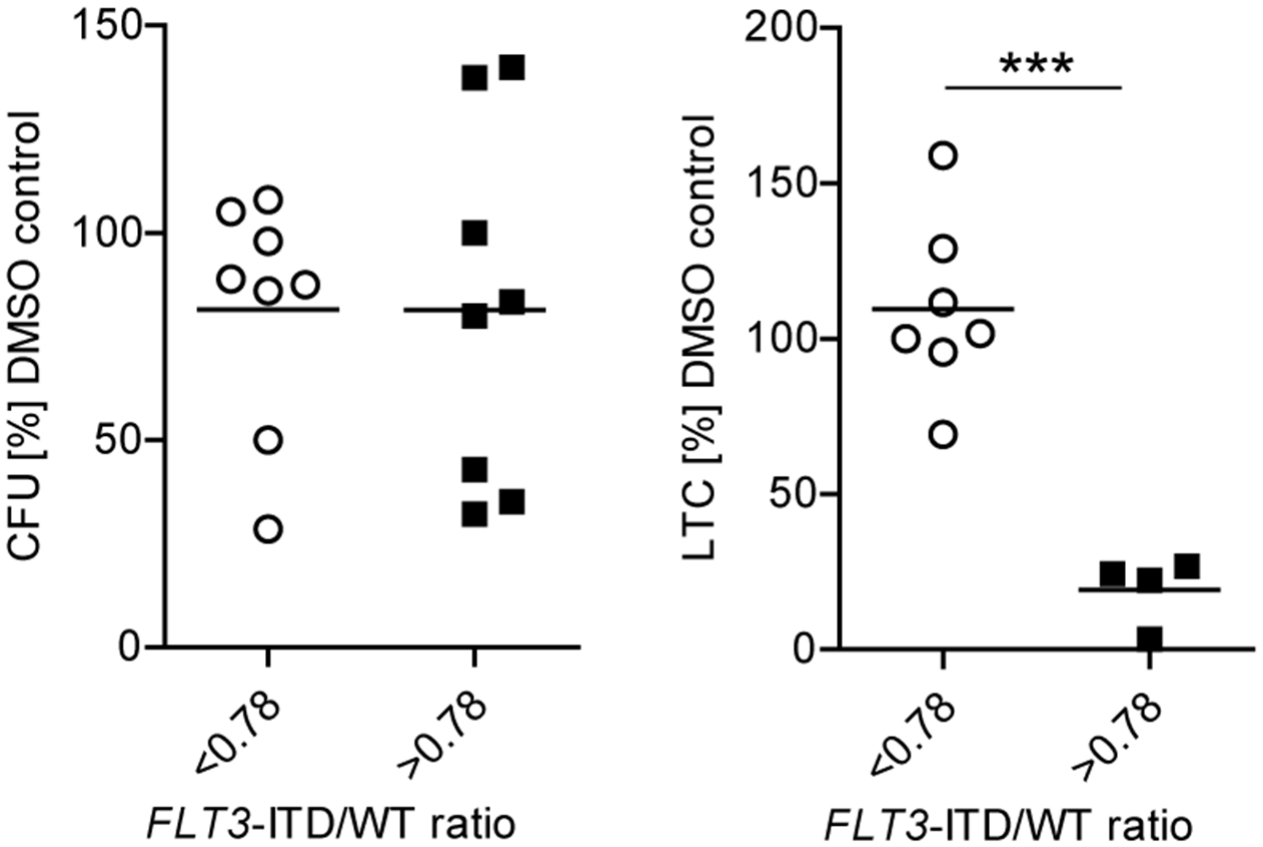
Sensitivity of primary *FLT3*-ITD LIC to creno is dependent on *FLT3*-ITD/WT ratio in the absence of *TET2* mutations Analysis of CFU (n = 16) and LTC (n = 11) capacity of *FLT3*-ITD/*TET2*wt samples in relation to *FLT3*-ITD/WT ratios after 4-day treatment with creno in the *in vitro* BM model.

Higher *FLT3*-ITD/WT ratios have been demonstrated to increase sensitivity towards TKI with published ratios ranging from 0.5-0.78 [44, 45]. Accordingly, in *FLT3*-ITD AML without *TET2* mutations only long-term LIC with a *FLT3*-ITD/WT ratio >0.78 were effectively targeted by creno while leukemic progenitors with a ratio <0.78 were not sensitive (Figure 5).

Importantly, the combination of AZA and creno effectively targeted *FLT3*-ITD LIC regardless of *FLT3*-ITD/WT ratios or concurrent mutations.

### AZA + creno inhibits engraftment capacity of *FLT3*-ITD LIC

**Figure 6:**
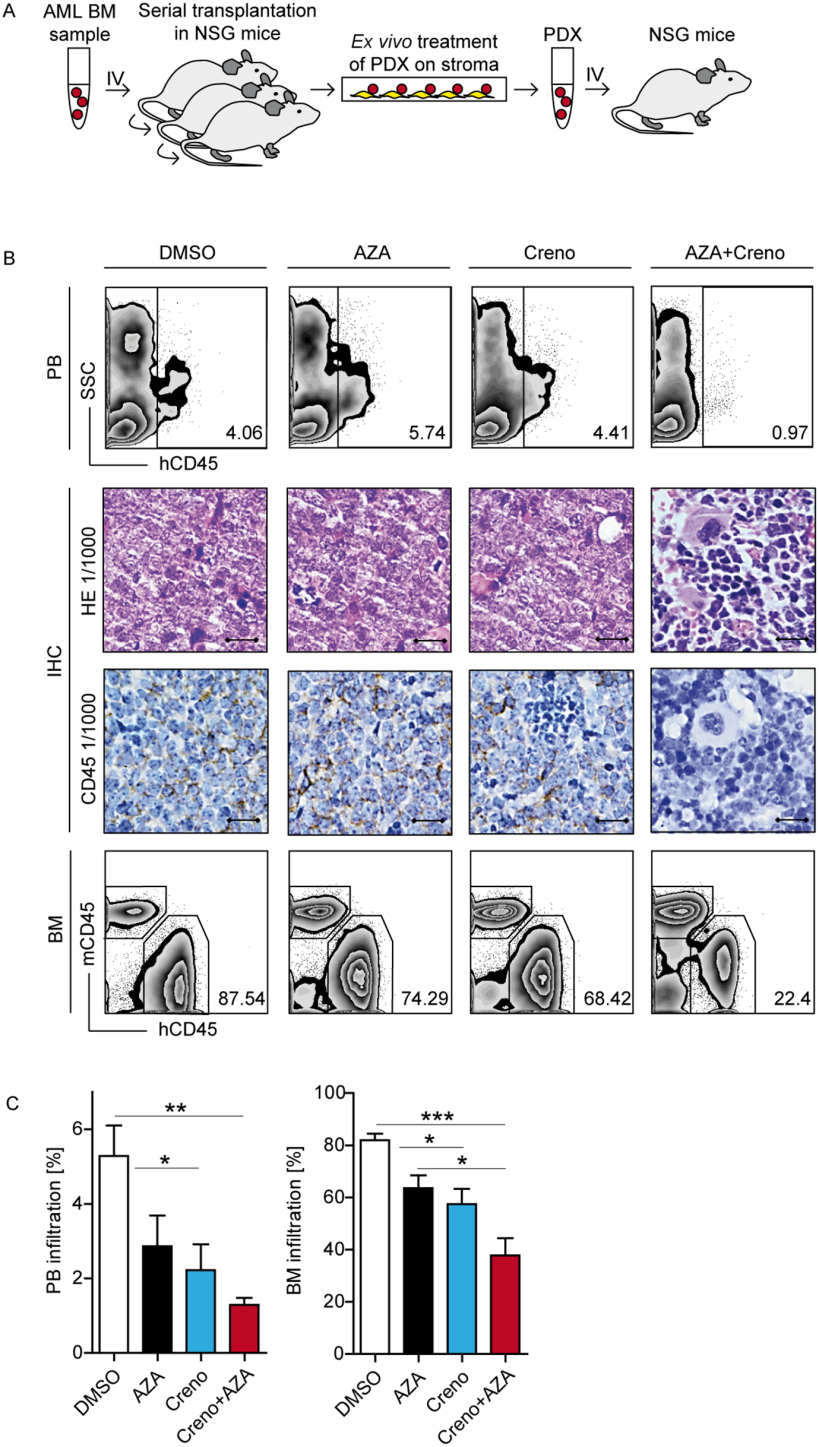
AZA + creno impairs engraftment capacity of *FLT3*-ITD LIC in a PDX mouse model Experimental design: PDX cells (AML sample ^#^361) were cultured *in vitro* on EL08-1D2 stroma and treated on day 1 with DMSO, 10 μM AZA, 100 nM creno or the combination thereof. PDX cells were harvested after 4 days and 2 x 10^5^ viable cells were injected IV into NSG mice (n= 20 per experiment) **(A)**. Representative images of *FLT3*-ITD/*NPM1*mut/*DNMT3A*mut PDX infiltration was analyzed by flow cytometry of PB (top) and BM (bottom) as well as immunohistochemistry (IHC) (middle; Scale bar, 10 μm) of femurs visualized by hematoxylin-eosin (H&E) staining or expression of human CD45 (hCD45) **(B)**. Statistical summary of PB and BM infiltration in NSG mice, mean of n=5 per condition ± SEM **(C)**.

To recapitulate our *in vitro* results with primary AML samples, we made use of a patient-derived xenograft (PDX) AML mouse model. PDX models faithfully mimic patient characteristics and thus are a powerful tool to define drug efficacy and resistance in leukemia [46, 47]. Primary *FLT3*-ITD AML samples were serially transplanted into NSG mice with reliable and reproducible engraftment for several passages, proving presence of LIC. Targeted resequencing of PDX cells demonstrated that driver mutations present in the founding clone of the original AML were preserved during passaging [46]. We tested the *in vivo* engraftment capacity of two separate *FLT3*-ITD-mutated PDX samples (Table 2) after *in vitro* treatment for 4 days with DMSO, creno, AZA or the combination of both (Figure 6A). Of note, our experimental read-out focused on the engraftment behavior of residual niche-protected LIC after drug treatment, which models the patient’s situation at post-remission. It is technically nearly impossible to model post-remission treatment with presence of dormant LIC *in vivo* (at least we are not aware of any such-like AML mouse model).

**Table 2:** PDX sample characteristics [46]

ID	Age	Sex	Disease stage	Cytogenetics	VAF of driver mutation in PDX (patient)			
					*FLT3*-ITD/WT ratio	*NPM1*	*DNMT3A*	*TET2*
AML361	40	F	Initial	Normal	0.31	0.48	0.47	wt
AML602	40	F	Relapse	Aberrant complex	0.39	0.39	wt	0.39

In the first PDX cohort (*FLT3*-ITD/*NPM1*mut/*DNMT3A*mut), engraftment was monitored by serial PB sampling. At 7 weeks post-transplant, PDX cells (mCD45^-^/hCD45^+^/hCD33^+^) were detected in PB of all mice in the DMSO cohort (mean 5.28%; range 3.33-7.14%), at which point all mice were sacrificed. PDX cells in PB were also detected in the AZA (mean 2.87%; range 1.16-5.74%), creno (mean 2,22%; range 0.85-4.41%) and AZA + creno (mean 1.29%; range 0.97-1.81%) groups. Strikingly, PDX infiltration of BM as assessed by FACS and IHC was significantly reduced in mice transplanted with AZA + creno-treated *FLT3*-ITD cells compared to DMSO control or AZA alone (Figure 6B and 6C).

**Figure 7:**
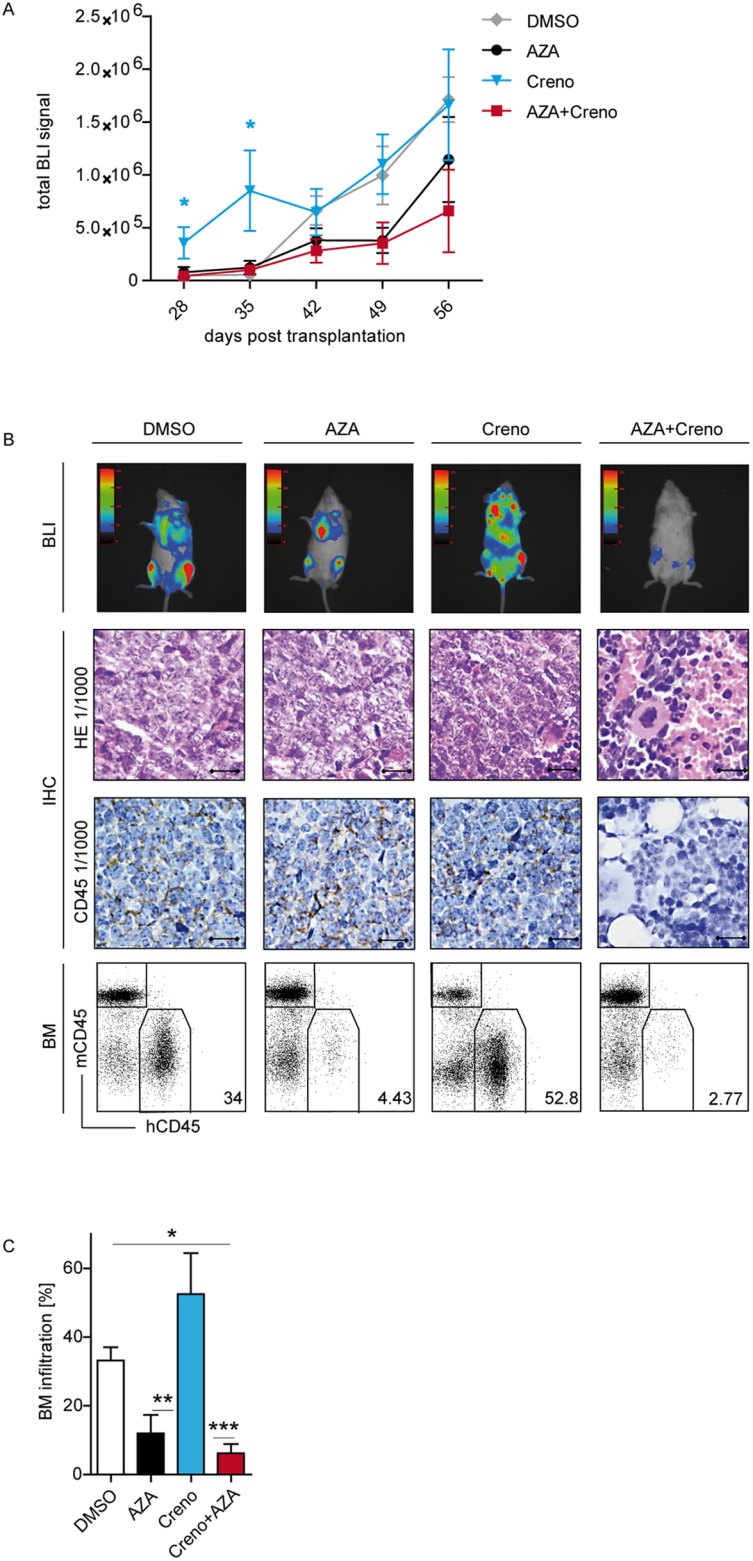
*In vivo* expansion of residual *FLT3*-ITD/*NPM1*mut/*TET2*mut PDX cells is enhanced after crenolanib monotherapy PDX cells (AML sample ^#^602) were cultured *in vitro* on EL08-1D2 stroma and treated with DMSO, 10 μM AZA, 100 nM creno or the combination thereof added on day 1. PDX cells were harvested on day 4 and 2 x 10^5^ viable cells were injected IV into NSG mice (n= 20 per experiment). AML infiltration in NSG mice was monitored by noninvasive BLI *in vivo* imaging. Time course of bioluminescence signals in NSG cohorts, mean of n=5 per condition ± SEM **(A)**. Representative BLI one day before sacrifice are shown (**B**, top). IHC of femurs (B, middle) visualized by H&E staining and expression of hCD45. Scale bar, 10 μm. FACS plots of BM (B, bottom). Statistical summary of BM infiltration in PDX transplanted NSG mice (mean of n=5 per condition ± SEM) **(C)**.

In the second PDX cohort (*FLT3*-ITD/*NPM1*mut/*TET2*mut) stable expression of luciferase enabled monitoring of engraftment by luciferin-induced bioluminescence. BLI signals were first detected in the BM of extremities and sternum 4 weeks post-transplantation. Notably, during the first 35 days, leukemic expansion of *FLT3*-ITD/*NPM1*mut/*TET2*mut cells was significantly accelerated in the creno treatment group compared to DMSO controls (Figure 7A). After 8 weeks, leukemia was spread over the whole body in 5/5 mice in the DMSO group, in 3/5 mice in the AZA group and in 4/5 mice in the creno group, at which point all the mice were sacrificed. In the AZA + creno group, only one mouse was highly infiltrated and no BLI signal was detectable in 2/5 mice until the end of BLI measurement (Figure 7B, Supplementary Figure 6). We verified BLI findings by FACS analysis and IHC. Corresponding to our CFC data (Figure 4C), BM infiltration by *FLT3*-ITD/*NPM1*mut/*TET2*mut PDX cells was increased in the creno cohort compared to DMSO control, while it was decreased in the AZA group and further significantly reduced in the AZA + creno cohort (Figure 7B and 7C), in which normal BM architecture was preserved as assessed by IHC (Figure 7B). Thus, our PDX data confirm that *TET2* mutations confer resistance and growth advantage in response to crenolanib as a single agent.

Thus, as proven by impaired engraftment of two different *FLT3*-ITD mutated PDX samples in NSG mice, the combination of AZA and creno abrogates stromal protection and effectively diminishes residual LIC in *FLT3*-ITD AML irrespective of cooperating genetic mutations. Of note, the two PDX samples differed in their cytogenetics, with the *TET2*-mutated sample showing a complex aberrant karyotype while the *DNMT3A*-mutated sample exhibited a normal karyotype (Table 2). To our knowledge, there is no data indicating an influence of aberrant karyotypes on the efficacy of TKI in *FLT3*-ITD AML. However, most studies on FLT3-TKI have been performed in normal karyotype AML which is by far the most common cytogenetic subgroup [48]. Thus, we cannot completely exclude that the different cytogenetics may have an influence on our results, although this seems unlikely.

### AZA modulates gene expression pattern of engaged LIC and niche cells

**Figure 8:**
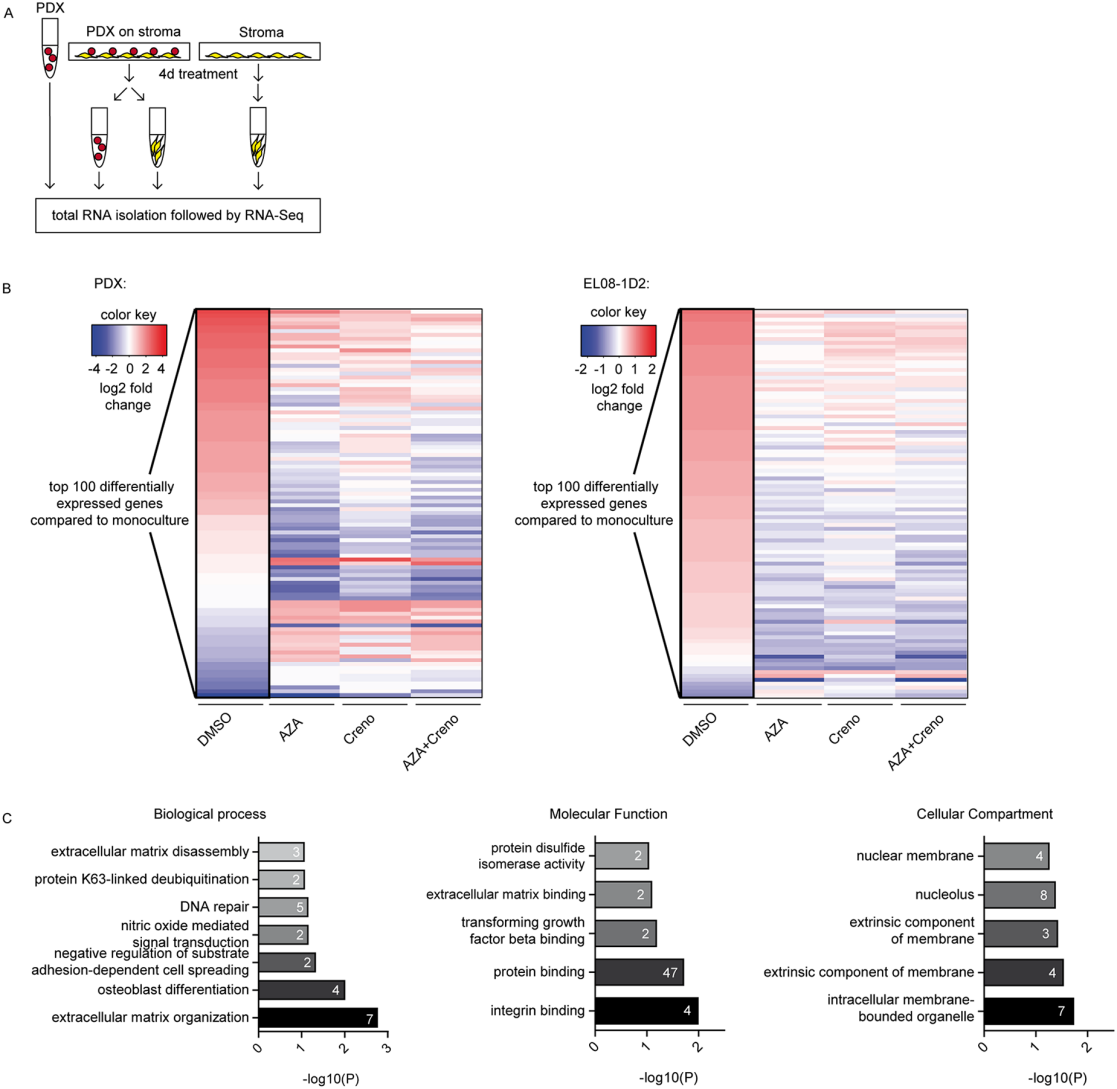
RNA sequencing analyses of *FLT3*-ITD PDX cells and EL08-1D2 stromal cells reveal changes after treatment in co-culture Experimental design: Total RNA was isolated from PDX and EL08-1D2 cells, either in monoculture (untreated) or co-cultured and treated on day 1 with DMSO, 10 μM AZA, 100 nM creno or the combination and cultured for 4 days *in vitro*. Four replicates were processed and analyzed for each condition **(A)**. First, we estimated the differential gene expression (log2 fold change, standard DESeq2 analysis) between stroma and LIC alone versus co-cultured cells. Then, log2 fold changes for the 100 most differentially expressed genes were ranked in ascending order, to visualize how treatment with AZA, creno or AZA + creno affected interaction of LIC and stroma in comparison to DMSO controls. Red and blue colors display up- and downregulation (log 2-fold change) of the 100 most differentially expressed genes in comparison to monoculture controls **(B)**. GO annotation for PDX genes. The number of significantly (p < 0.05) enriched genes per GO category are indicated **(C)**.

To delineate in how far stromal protection of *FLT3*-ITD LIC is modulated by treatment, we evaluated the individual gene expression pattern by RNAseq of co-cultured *FLT3*-ITD/*NPM1*mut/*DNMT3A*mut PDX cells (AML sample #361, Table 2) with EL08-1D2 cells after *in vitro* treatment with DMSO, AZA, creno or the combination (Figure 8A).

Strikingly, interaction of PDX cells with EL08-1D2 cells in co-culture altered the gene expression pattern of both cell types compared to monoculture control cells (Figure 8B, up- and downregulated genes in the DMSO lane), illustrating active engagement. Pathway analyses and functional GO annotation of the top 100 differentially expressed genes between DMSO-control and treatments revealed on the PDX side enrichment of genes in extracellular-matrix-receptor interaction (Figure 8C, Table 3, Supplementary data). On the EL08-1D2 side, analysis highlighted genes involved in signaling pathways shown to promote a leukemic microenvironment, e.g. NF-kappB, HIF-1 and TNF signaling (Table 4, Supplementary data). Of note, this gene expression pattern was partially reversed through treatment with AZA alone and in combination with crenolanib while crenolanib alone induced a less visible, slightly different pattern (Figure 8B). The emerging differences in gene expression pattern provide further evidence that AZA effectively impairs LIC-niche interaction and thus overcomes stromal resistance against crenolanib treatment.

**Table 3:** Pathway analysis in PDX cells

KEGG Pathways	Significantly enriched genes
hsa04512:ECM-receptor interaction	*CD36, COL5A2, SPP1*

**Table 4:** Pathway analysis in niche cells

KEGG Pathways	Significantly enriched genes
mmu04620:Toll-like receptor signaling pathway	*Tab2, Chuk, Cxcl10, Lbp*
mmu04064:NF-kappa B signaling pathway	*Tab2, Chuk, Lbp*
mmu04066:HIF-1 signaling pathway	*Serpine1, Egln1, Vegfa*
mmu04668:TNF signaling pathway	*Tab2, Chuk, Cycl10, Lbp*
mmu05200:Pathways in cancer	*Arhgef12, Chuk, Rock1, Egln1, Vegfa*
mmu04270:Vascular smooth muscle contraction	*Arhgef12, Npr2, Rock1*

## DISCUSSION

In order to cure AML, any effective therapy must eliminate the chemo-resistant, quiescent and self-renewing LIC enriched within the CD34^+^CD38^-^ population of the BM. Eradication of LIC in *FLT3*-ITD AML by FLT3-TKI has thus far remained an elusive goal. Here, using multiparameter flow cytometry in combination with targeted sequencing, we detected *FLT3*-ITD within the Lin^-^/CD33^(+)^/CD45^dim^/CD34^+^CD38^-^ LIC compartment beginning at the level of HSC or MPP, i.e. in the same early compartment as mutations in *NPM1*, *DNMT3A* or *TET2* inferred by NGS data. This is in line with recent data demonstrating that either *DNMT3A* or *TET2* mutations together with *FLT3*-ITD alone are sufficient to transform murine HSC and induce AML in mouse models [49, 50]. Thus, although *FLT3*-ITD may be subclonal in some leukemias and is sometimes lost at relapse, our data ascertain the validity of *FLT3*-ITD as a TKI target to eliminate LIC.

Nevertheless, the highly FLT3-selective TKI crenolanib alone could not effectively target stroma-protected *FLT3*-ITD LIC in our *in vitro* BM model. Although LIC with higher *FLT3*-ITD/WT ratios were more sensitive to creno, this only held true for samples without *TET2* mutations. *FLT3*-ITD/*TET2*mut LIC were completely resistant to creno as a single agent in the presence of stroma and expanded upon TKI treatment with CFC results corroborated by our PDX transplant data. In support of our primary human data, TKI insensitivity has been observed in *FLT3*-ITD/*TET2*^*-/-*^ murine progenitors as well [49]. This observation warrants confirmation in a larger clinical cohort as it may have important implications for tailoring of TKI therapy in *FLT3*-ITD AML.

Since *IDH* mutations inhibit *TET2* function and induce a similar leukemic signature as *TET2* mutations [51], it is tempting to speculate that *IDH* mutations may also confer resistance to TKI in *FLT3*-ITD AML. Unfortunately, we were unable to investigate this due to the limited number of *IDH*-mutated AML samples in this study.

Strikingly, addition of AZA to creno effectively overcame niche protection of *FLT3*-ITD LIC *in vitro*, resulting in significantly reduced expansion in CFC assays despite concurrent *NPM1, DNMT3A* or *TET2* mutations. In addition, engraftment ability of *in vitro*-treated *FLT3*-ITD PDX cells was severely impaired, providing further evidence we are targeting *FLT3*-ITD LIC with long-term proliferative potential. Translated to the clinical setting, this would suggest that the combination of AZA + creno could be employed as maintenance therapy to suppress residual LIC.

Conflicting data has been reported on the correlation between clinical response to AZA and the presence of *DNMT3A* or *TET2* mutations in retrospective analyses, but data from prospective trials have not been reported thus far [16, 17, 23, 24, 52, 53]. In our cohort directly testing AZA on primary *FLT3*-ITD LIC, the presence of *NPM1*, *DNMT3A* or *TET2* mutations did not confer a better response to AZA alone, arguing against a direct link between AZA efficacy and epigenetic mutations. Furthermore, despite many efforts, a clear correlation between DNA demethylation and response to AZA has not been able to be demonstrated. The fact that AZA is incorporated to a large part into RNA (up to 90%) and only to a smaller percentage into DNA [25, 54], suggests that the observed effect of AZA on stromal resistance of *FLT3*-ITD LIC may also be mediated via the RNA level and not only by DNA demethylation.

Indeed, our RNA-seq data suggest that several pathways involved in the complex interaction between LIC and their niche were actively disrupted by AZA that warrant further investigation. Crosstalk between LIC and the niche is not dependent on one single mechanism but is a dynamic process mediated by diverse mechanisms including cellular and soluble mediators. Surely, we were not able to depict the entire complexity of stromal TKI resistance of LIC in our *in vitro* co-culture assays. However, at least in our experimental setup, TKI resistance was not mediated by reactive FL secretion as has been described previously [19] or niche-derived soluble factors, but was dependent on direct association between LIC and niche cells. To analyze and validate each of these pathways was beyond the scope of this project but will be performed in future work. In itself, the diversity of RNA and DNA-dependent pathways by which AZA may exert its anti-leukemic activity argues that the multimodality of mechanisms is key to effectively overcoming stromal resistance so as to enable creno to specifically target *FLT3*-ITD LIC in their niche. It therefore seems unlikely that targeting a singular pathway involved in stromal protection may recapitulate this effect.

To our knowledge, this is the first work studying the effect of TKI and AZA on different mutational genotypes in primary human *FLT3*-ITD AML. We demonstrate that efficacy of TKI therapy in *FLT3*-mutated AML is highly dependent on the mutational landscape within each leukemia and provide a valuable framework for designing rational combination therapy in *FLT3*-ITD AML. The recently reported encouraging results of the RATIFY trial show that combining the unselective TKI midostaurin with intensive chemotherapy results in a survival benefit for *FLT3-*ITD AML patients, but it is uncertain how much of this effect is due to inhibition of FLT3 as opposed to off-target effects of midostaurin [55]. Our data suggest that testing patients with *FLT3*-ITD AML for *TET*2 mutations should be considered before initiation of more selective FLT3-TKI therapy and that the combination of AZA + TKI could be employed as a means to suppress residual LIC.

## MATERIALS AND METHODS

### Cell lines and reagents

MV4-11, MOLM-13 and RS4;11 cell lines were obtained from and propagated as suggested by the German Collection of Microorganisms and Cell Cultures (DMSZ), were authenticated by DMSZ by DNA typing and PCR analysis as well as cytogenetic testing, and passaged for <6 months after receipt. EL08-1D2 stromal cells were used up to passage 12 and cultured as described [9]. Ba/F3_*FLT3*-WT and Ba/F3_*FLT3*-ITD cells were generated as described previously [56]. All cytokines were purchased from R&D Systems. Creno (Selleckchem, Munich, Germany) and AZA (Celgene Corp, Munich, Germany) were freshly prepared directly before use following the manufacturers’ instructions.

### Bone marrow samples

BM samples were obtained from newly diagnosed AML patients recruited to the German AMLSG trials or from the Munich Leukemia Laboratory (MLL). Written informed consent in accordance with the Declaration of Helsinki was obtained from all patients according to protocols approved by the Ethics Committee of the TUM. Samples were screened for presence of *FLT3*-ITD, *DNMT3A, NPM1*, *TET2* and *IDH1/2* mutations as described [48]. Mononuclear cells (MNC) were isolated from BM using Bicoll separating solution and stored in liquid nitrogen until use. Patient sample characteristics are summarized in Table 1.

### Flow cytometric sorting (FACS) of leukemic blast, progenitor and stem cell compartments

MNC from *FLT3*-ITD AML BM samples were stained for lineage markers using biotinylated antibodies: CD4 (RPA-T4; Biolegend), CD8a (RPA-T8; Biolegend), CD19 (HIB19; Biolegend), CD41 (MEM-06; Sigma), CD235alpha (HIR2; eBioscience), CD56 (B159; BD Pharmingen). Cells were then stained with the following fluorochrome-conjugated antibodies: CD34-FITC (581; BD Pharmingen), CD90-PE (5e10; BD Pharmingen), CD33-PC5.5 (D3HL60, Beckmann Coulter), CD45RA-APC Cy7 (H1100; BD Pharmingen), Streptavidin-eFluor 450 (eBioscience), CD38-APC (HB7; BD Pharmingen), CD45-APC-Cy7 (2D1; BD Pharmingen). PI was added as live/dead marker. Cell sorting was performed on a FACS Aria II (Becton Dickinson, Heidelberg, Germany). Sorting purity of >98% was routinely obtained.

### gDNA Isolation of FACS-sorted stem cell compartments

gDNA was isolated from sorted AML subpopulations using the ZR Viral DNA Kit™ (Zymo Research, Freiburg, Germany) for <2 x 10^5^ cells and the QIAamp DNA Micro Kit (Qiagen, Hilden, Germany) for >2 x 10^5^ cells according to the manufacturers’ instructions.

### Targeted sequencing of AML bulk and sorted subpopulations

Molecular alterations in the gDNA from AML bulk samples (AML samples #1-10) were evaluated by targeted sequencing using the TruSight Myeloid assay (Illumina, Chesterford, UK) which covers the following 54 genes or gene hotspots related to myeloid neoplasms: *BCOR*, *BCORL1*, *CDKN2A*, *CEBPA*, *CUX1*, *DNMT3A*, *ETV6*, *EZH2*, *IKZF1*, *KDM6A*, *PHF6*, *RAD21*, *RUNX1*, *STAG2* and *ZRSR2* and oncogenic hotspots of *ABL1*, *ASXL1*, *ATRX*, *BRAF*, *CALR*, *CBL*, *CBLB*, *CBLC*, *CDKN2A*, *CSF3R*, *FBXW7*, *FLT3*, *GATA1*, *GATA2*, *GNAS*, *HRAS*, *IDH1*, *IDH2*, *JAK2*, *JAK3*, *KIT*, *KRAS*, *MLL*, *MPL*, *MYD88*, *NOTCH1*, *NPM1*, *NRAS*, *PDGFRA*, *PTEN*, *PTPN11*, *SETBP1*, *SF3B1*, *SMC1A*, *SMC3*, *SRSF2*, *TET2*, *TP53*, *U2AF1* and *WT1*. For preparation of target enrichment libraries, 50 ng of genomic DNA were used and prepared as recommended in the manufacturers’ protocol (TruSight Myeloid Sequencing Panel Reference Guide 15054779 v02, Illumina). Samples were paired-end sequenced (2x225 bp) on a MiSeq sequencer using MiSeq Reagent Kits V3 (Illumina). Sequence data alignment of demultiplexed FastQ files, variant calling and filtering was done using the Sequence Pilot software package (JSI medical systems GmbH, Germany) with default settings and a 5% variant allele frequency (VAF) mutation calling cut-off. Detection of large insertions and deletions was performed using PINDEL algorithm following BWA-MEM mapping with default settings [57, 58]. Human genome build HG19 was used as reference genome for mapping algorithms.

Analysis in gDNA extracted from sorted AML subpopulations (AML samples #2-4, 6-7, 9) was done using targeted resequencing based on individual amplicons generated specifically for the affected regions. Deep sequencing was performed on MiSeq (Illumina) or IonTorrent PGM (Thermo Fisher Scientific, Darmstadt, Germany) platforms with a minimum coverage of 10,000 reads.

### Cell viability assays

Dose-dependent cytotoxicity was determined by colorimetric quantification of dimethyl-thiazole diphenyl tetrazolium bromide (MTT; Promega) according to the manufacturer’s instructions. To determine cell death, cells were washed and stained in Annexin/PI buffer containing 1 M Hepes, 2.5 M NaCl, 1.62 mM CaCl_2_, and APC-Annexin V (BD Biosciences) and PI was added. Flow cytometric analyses were performed on a CyAn ADP Lx P8 (Beckman Coulter). Data were processed with FlowJo software (TreeStar Inc, Ashland, Oregon, USA).

### Western blot analysis

SDS-PAGE and immunoblotting were performed as previously described [9]. Cells were collected and washed in ice-cold PBS containing 1 mM Na2VO4 and shock frozen in liquid nitrogen. Cell pellets were thawed on ice and incubated in lysis buffer. After 15 minutes, lysates were sonicated. Cell debris was removed by centrifugation at full speed for 15 minutes at 4°C in a microcentrifuge. Supernatants were collected and the protein concentration was estimated using the DC Protein Assay Kit II following the manufacturers’ instructions. Protein absorption was measured at 750 nm using the ELx800 Universal Microplate reader and the Microplate Manager 5.2. software. 30 μg protein were taken up in loading dye, boiled at 95°C for 10 minutes and separated in 4-15 % Mini Protean TGX stain free gels by SDS-PAGE in 1X Running Buffer in an electrophoresis chamber. The gel was blotted onto PVDF membranes in transfer buffer in a wet-transfer device. After blocking in 5% BSA or milk in TBS-T, membranes were incubated overnight at 4°C with the following primary antibodies: FLT3/FLK2 (1:1000), pFLT3 (Tyr591) (1:1000), ERK1 (1:1000), pERK (1:1000), Stat5 (1:1000), pStat5(Tyr 694) (1:1000), AKT (1:1000), pAKT (Ser473) (1:1000), or ß-actin (1:2000). After washing with TBS-T, anti-rabbit or anti-mouse IgG ECL HRP-linked secondary antibodies were added for 20 minutes at RT. Membranes were washed with TBS and visualized on Kodak films using the enhanced chemiluminescence (ECL)-method (SuperSignal West). Signal intensity was analyzed using the ImageJ software. Antibodies were stripped off the membranes using Amido Black Stain solution.

### Culture of primary AML cells

MNC from *FLT3*-ITD BM samples were enriched for CD34^+^ cells by magnetic bead separation (Miltenyi Biotech Bergisch Gladbach, Germany). CD34^+^ cells were cultured on EL08-1D2 in serum-free medium (SFM) with 5 growth factors (5 GF): kit ligand (KL), Flt3-ligand (FL), thrombopoietin (TPO), interleukin-6 (IL-6) and interleukin-3 (IL-3) as described [9]. Cells were treated with creno (100 nM), DMSO or AZA (10 uM) on day 1 of the culture as indicated. After 4 days, CD34^+^ cells were harvested and assayed for short-term colony-forming units (CFU) and long-term culture-derived colony-forming cells (LTC) as described [9].

### PDX xenograft mouse model

Animal studies were performed in agreement with the Guide for Care and Use of Laboratory Animals published by the US National Institutes of Health (NIH Publication No. 85-23, revised 1996), in compliance with German law on the protection of animals, and with approval of the responsible regional authorities. NOD.C_g_-*Prkdc*^*scid*^ IL2rg^*tm1Wjl*^/Sz mice (NSG; The Jackson Laboratory, Bar Harbour, ME) were maintained at the animal facility of the TUM.

*FLT3*-ITD AML patient derived xenograft (PDX) cells and transgenic PDX cells which express firefly luciferase were generated by serial passaging in NSG mice as described [46]. PDX cells were cultured on EL08-1D2 cells and treated with DMSO, AZA, creno or both for 4 days *in vitro*. Cells were harvested and viable cells were enumerated by trypan blue exclusion. A total of 2 x 10^5^ viable cells (per mouse) in 200 μl PBS were injected into the tail vein of non-irradiated 6 to 16-week-old NSG mice. To monitor engraftment, peripheral blood (PB) samples were collected every other week beginning week 6 after PDX injection from the facial vein of NSG mice and analyzed by flow cytometry.

Transgenic PDX engraftment in NSG mice was repetitively monitored by bioluminescence *in vivo* imaging (BLI). Mice were narcotized and injected with D-luciferin (BIOMOL, Germany) intraperitoneally to activate recombinant luciferase. Images were taken using a charge-coupled device camera, equipped with an image intensifier for 10-120 seconds, bin size 2, gain 900 (OrcaII ER, Hamamatsu, Japan) as previously described [59]. The SimplePCI software (Hamamatsu) was used to display and quantify BLI signals.

Mice were sacrificed when PB sampling or BLI indicated systemic AML in the DMSO-treated mice. PDX cells were re-isolated from PB, femurs, tibiae and spines. After erythrocyte lysis, cells were stained with the following antibodies for flow cytometry: hCD45 APC-Cy7 (BD Pharmingen, 2D1), hCD34-FITC (BD Pharmingen, 581), hCD33-PC5.5 (Beckmann Coulter, D3HL60.251), mCD45-APC (eBioscience, 30-F11), Cell Trace Violet (Life Technologies). Flow cytometric analyses were performed on a CyAn ADP Lx P8 (Beckman Coulter, Krefeld, Germany). Data were processed with FlowJo software (TreeStar, Inc).

### Immunhistochemistry

Tissue sections were deparaffinized. Antigen retrieval was carried out by pressure cooking in citrate buffer (pH 6) for 7 minutes. Anti-human CD45 was detected after 1-hour incubation at room temperature by the Mouse on Mouse kit (Abcam) according to the manufacturer's protocol. Slides were evaluated using an Olympus BX53 microscope (x100 objective, Olympus, Germany) and images taken with an Olympus DP26 camera (Olympus).

### RNA sequencing

Total RNA was isolated from murine EL08-1D2 and human PDX cells using RNeasy Mini Kit (Qiagen). RNA sequencing (RNA-seq) library preparation and data processing was performed according to the Unique Molecular Identifiers (UMI) sequencing protocol, a modified bulk single-cell RNA barcoding and sequencing (SCRB)-seq protocol [60].

Briefly, cDNA libraries were prepared from 5 ng RNA per sample. Unique molecular identifiers (UMIs) and sample barcodes were introduced during reverse transcription. Next, cDNA of all samples were pooled, unincorporated primers were digested with Exonuclease I and pre-amplified by single-primer PCR (12 cycles). After quality control of amplified cDNA, the sequencing library was constructed from 1 ng full-length cDNA using the Nextera XT Kit (Illumina). To enrich for barcoded 3’ ends in the Nextera XT library, a custom i5 primer was used. Library pools were sequenced on an Illumina HiSeq1500 on a rapid flow-cell with paired-end layout. The first read contained the sample barcode and the UMI sequences (16 cycles), while the second read contained the cDNA fragment (50 cycles). Note that UMI sequencing is a 3' method to count the number of unique transcripts. This procedure has the advantage that not the whole gene length has to be covered and a smaller number of reads compared to full-length RNA sequencing methods is sufficient [60, 61].

All samples were mapped against concatenated human (hg19) and mouse (mm10) genomes and gene features were quantified using Ensembl gene models (v75). Gene expression of PDX samples (reads mapped to human) and EL08 samples (reads mapped to mouse) was analyzed separately.

The total number of mapped reads and the number of reads per species (human or mouse) were calculated for each sample. In addition, the percentage of reads per species (human or mouse) was calculated. To calculate the number of expressed genes per sample, UMI counts were normalized such that each sample had a total number of 1 million counts. The comparability of UMIs across experiments was checked with a box plot of log-transformed UMI values. The minimum UMI greater than 0 from each experiment was added to each value to avoid log(0). A gene was defined as expressed if the UMI count/million was greater or equal than 1. For PDX samples, only reads mapped to human, for EL08 samples, only reads mapped to mouse were considered.

Gene expression analysis and visualization was done with R version 3.2.0 on a x86_64-pc-linux-gnu (64-bit) platform under Ubuntu 14.04.2 LTS. Differential gene expression analysis of PDX and EL08-1D2 in co-culture (DMSO) compared to PDX and EL08-1D2 alone (monoculture controls) was performed using DESeq2 (Supplementary Data). DESeq2 uses library-size-corrected read count data to find differentially expressed genes and is an error model based on the negative binomial distribution for the read counts. For gene expression analysis, the fits of the negative binomial with a generalized linear model were analyzed. Coefficients (interpreted as the log2 fold changes) were tested using the Wald test. The false discovery rate according to Benjamini-Hochberg was used for multiple testing corrections [62]. Each co-culture cohort (AZA, creno, AZA + creno) was then compared to respective DMSO cohorts and a log2 fold change for each gene and comparison was calculated (Supplementary Data, “Comparison 1”). Further, for each treatment condition (DMSO, AZA, creno, AZA + creno), log2 fold changes were compared to monoculture controls (Supplementary Data, “Comparison 2”). Those hundred genes with the highest average gene expression difference (“top 100 differentially expressed genes”) were sorted by log2 fold change according to the DMSO sample in ascending order as visualized in a heatmap. Finally, enriched gene ontology and KEGG pathways analysis was performed on the “top 100 differentially expressed genes” (S[Supplementary-material SD7 SD8]upplementary Data, “top 100 genes”).

### Statistical analysis

Statistical analyses were performed by Student’s t-test or repeated measures ANOVA using GraphPad Prism software (GraphPad Inc, La Jolla, CA). Linear regression analyses using Greenhouse-Geisser correction was performed in SPSS 24 software (IBM). *P* values are presented in the figures where a statistically significant difference was found: ^*^, *P* <.05; ^**^, *P* <.01; ^***^, *P* <.001.

## SUPPLEMENTARY MATERIALS FIGURES AND TABLE

















## Abbreviations

AML: acute myeloid leukemia
BLI: bioluminescence imaging
BM: bone marrow
CFC: colony-forming cell assay
CFU: colony-forming unit
LIC: leukemia-initiating cell
LTC: long-term culture assay
MNC: mononuclear cells
PDX: patient-derived xenograft
VAF: variant allele frequency
