# Supplementary material for: Azacitidine combined with the selective FLT3 kinase inhibitor crenolanib disrupts stromal protection and inhibits expansion of residual leukemia-initiating cells in FLT3-ITD AML with concurrent epigenetic mutations
Source: Oncotarget. 2017 Oct 16;8(65):108738–59. doi: 10.18632/oncotarget.21877 (PMC5752477; doi:10.18632/oncotarget.21877)
Supplement: Supplementary file 1 [file oncotarget-08-108738-s001.pdf]

Azacitidine combined with the selective FLT3 kinase inhibitor crenolanib disrupts stromal protection and inhibits expansion of residual leukemia-initiating cells in *FLT3*-ITD AML with concurrent epigenetic mutations

SUPPLEMENTARY MATERIALS

Supplementary Table 1: Analysis of co-occurring mutations in leukemic stem/progenitor cell compartments of *FLT3*-ITD^{+} AML BM samples at diagnosis

ID	Mutation	BM compartment					
		Blasts	Progenitors	Stem cells	MLP	MPP	HSC
#2	FLT3-ITD/WT ratio	0.72	3.9	11.3	12.9	39.2	1.8*
	NPM1mut	pos	pos	pos	pos	pos	pos*
#3	FLT3-ITD/WT ratio	0.35	7.7	25.1	31.5	1.3	0.11
	NPM1mut	pos	pos	pos	pos	pos	pos*
	RAD21mut VAF [%]	55%	62%	61%	60%	60%	45%
#4	FLT3-ITD/WT ratio	1.04	0.83	0.74	0.71	0.07	n.a.
	NPM1mut	wt	wt	wt	wt	wt	n.a.
	TET2mut VAF [%]	54%	56%	56%	56%	48%	n.a.
	DNMT3Amut VAF [%]	51%	51%	48%	51%	failed	n.a.
#6	FLT3-ITD/WT ratio	0.25	n.a.	n.a.	n.a.	n.a.	n.a.
	NPM1mut	pos	n.a.	n.a.	n.a.	n.a.	n.a.
	TET2mut VAF [%]	55%	22%	n.a.	failed	n.a.	n.a.
#7	FLT3-ITD/WT ratio	0.93	0.02	0.003	0	0*	n.a.
	NPM1mut	pos	pos	pos	pos	pos*	n.a.
	IDH1mut VAF [%]	45%	47%	43%	43%	0.5%	n.a.
	CEBPAmut VAF [%]	40%	30%	34%	21%	16%	n.a.
#9	FLT3-ITD/WT ratio	1.05	failed	1.33	0.5	n.a.	n.a.
	NPM1mut	pos	pos	pos	pos	n.a.	n.a.
	TET2mut VAF [%]	43%	40%	46%	44%	n.a.	n.a.
	DNMT3Amut VAF [%]	20%	12%	9%	25%	n.a.	n.a.
#10	FLT3-ITD/WT ratio	0.98	0.77	n.a.	n.a.	n.a.	n.a.
	NPM1mut	pos	pos	n.a.	n.a.	n.a.	n.a.
	TET2mut VAF [%]	51%	52%	n.a.	n.a.	n.a.	n.a.

Pos indicates positive;*, weak signal; n.a., not available (e.g. population not present in sort); VAF [%], variant allele frequency.

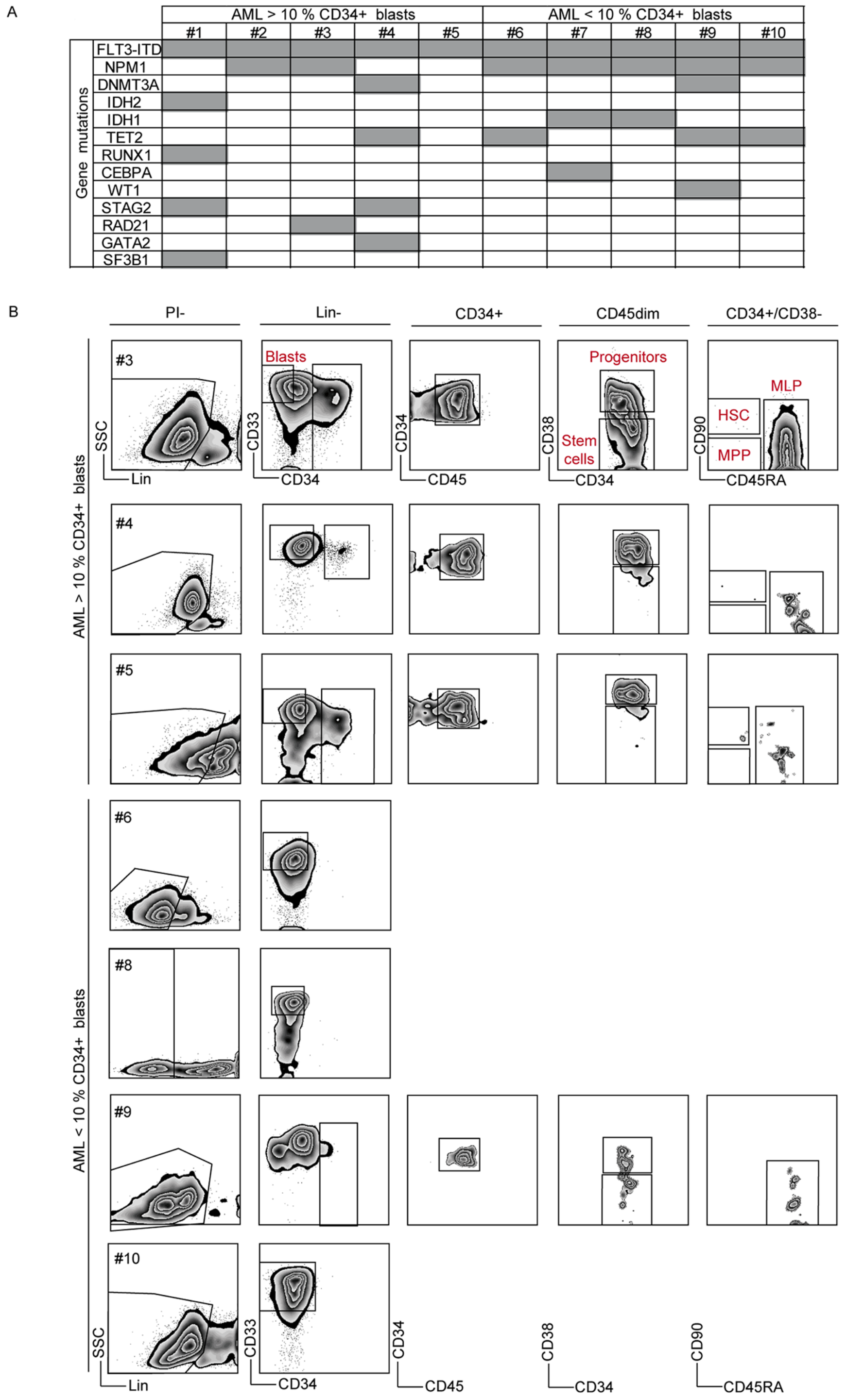

Supplementary Figure 1: Analysis of *FLT3*-ITD and cooperating gene mutations in AML bulk and leukemic stem/progenitor compartments. Mutational landscape of 10 unfractionated *FLT3*-ITD AML BM samples with CD34 blast expression >10% or <10% was assessed by targeted sequencing using the TruSight Myeloid assay (Illumina, Chesterford, UK) which covers the following 54 genes or gene hotspots related to myeloid neoplasms **(A)**. Mononuclear cells (MC) from CD34$^+$ and CD34$^-$ *FLT3*-ITD AML BM samples were separated by flow cytometric cell sorting. Blasts (Lin$^-$/CD33$^+$/CD34$^-$), committed progenitors (Lin$^-$/CD33$^{(+)}$/CD45^{dim}/CD34$^+$CD38$^+$), and early stem cell compartments (Lin$^-$ /CD33$^{(+)}$/CD45^{dim}/CD34$^+$CD38$^-$) i.e. multilymphoid progenitors (MLP; CD45RA$^+$), multipotent progenitors (MPP; CD45RA$^-$CD90$^-$) and hematopoietic stem cells (HSC, CD45RA$^-$CD90$^+$) **(B)**.

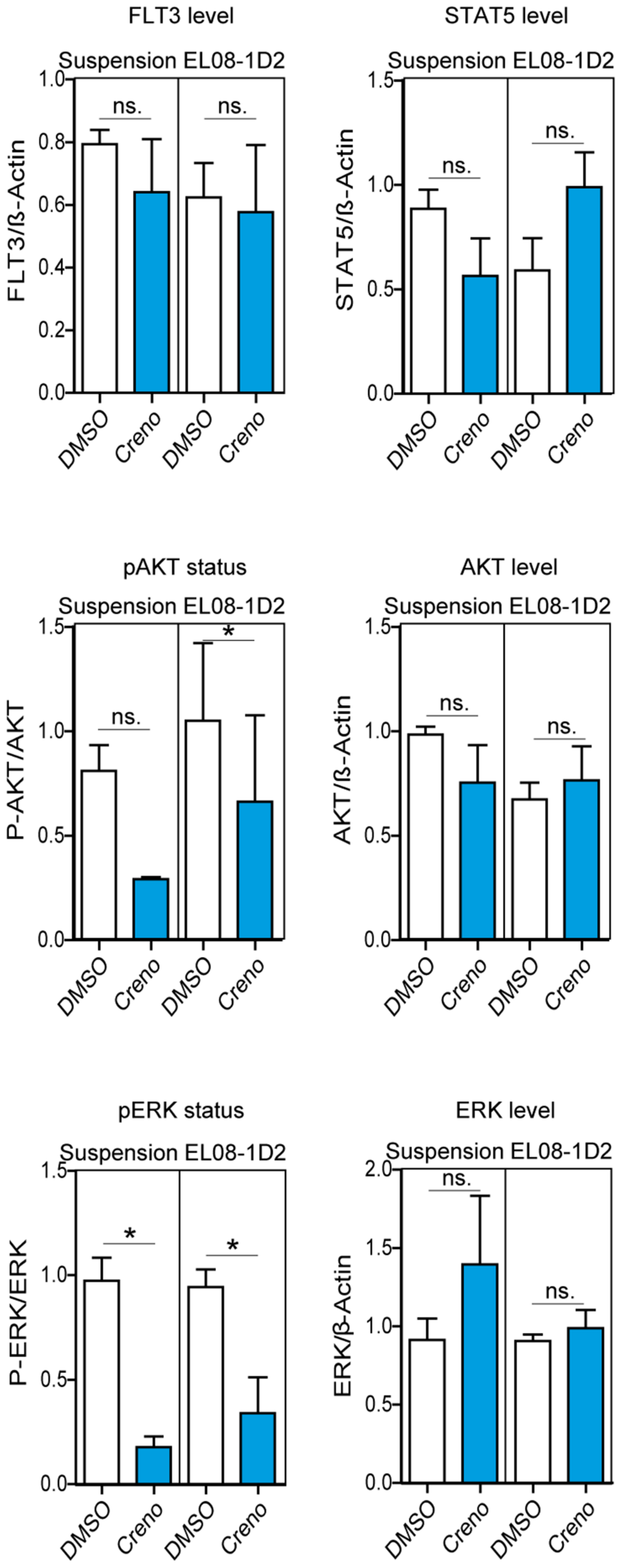

Supplementary Figure 2: Stromal TKI resistance is independent of *FLT3*-ITD signaling. *FLT3*-ITD signaling was analyzed by immunoblotting of Ba/F3_*FLT3*-ITD cells in suspension or in contact with stroma after treatment with DMSO or creno for 1h. Signal intensities were quantified using Image J software. Results are shown as mean of n=3 independent experiments ± SEM.

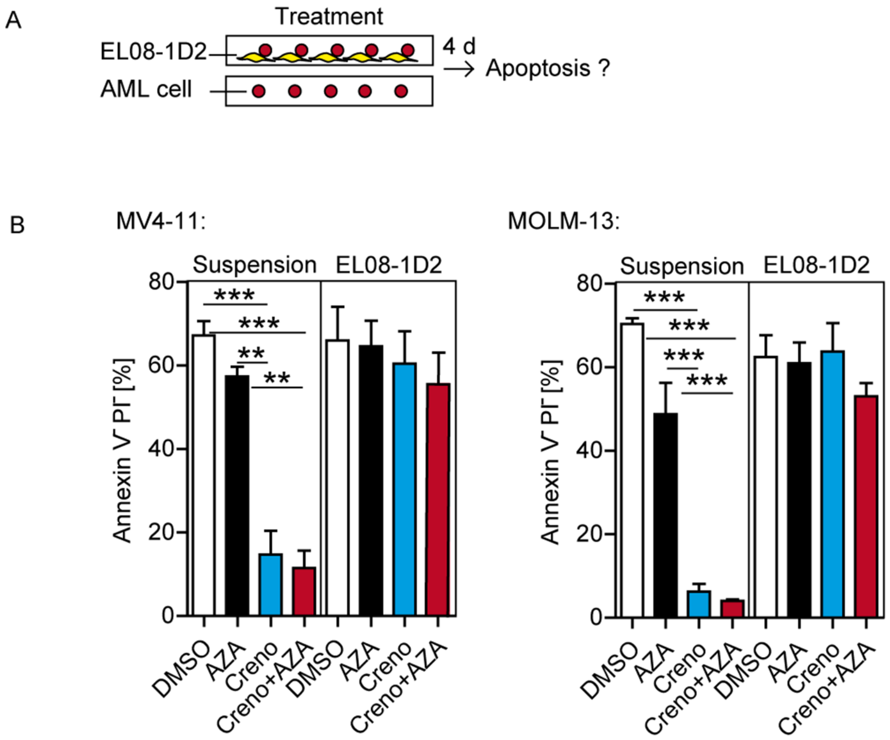

Supplementary Figure 3: Efficacy of creno as single agent and in combination with AZA in FLT3-ITD^{+} AML cell lines. MV4-11 and MOLM-13 cells were cultured in suspension or on stromal EL08-1D2 layers for 4 days. Cultures were treated with DMSO, AZA (10 µM), creno (100 nM) or in simultaneous combination. Induction of cell death was measured by Annexin V/PI flow cytometry. Experimental design **(A)**. Results for MV4-11 (left panel) and MOLM-13 (right panel) cells are shown as mean of three independent experiments ± SEM.

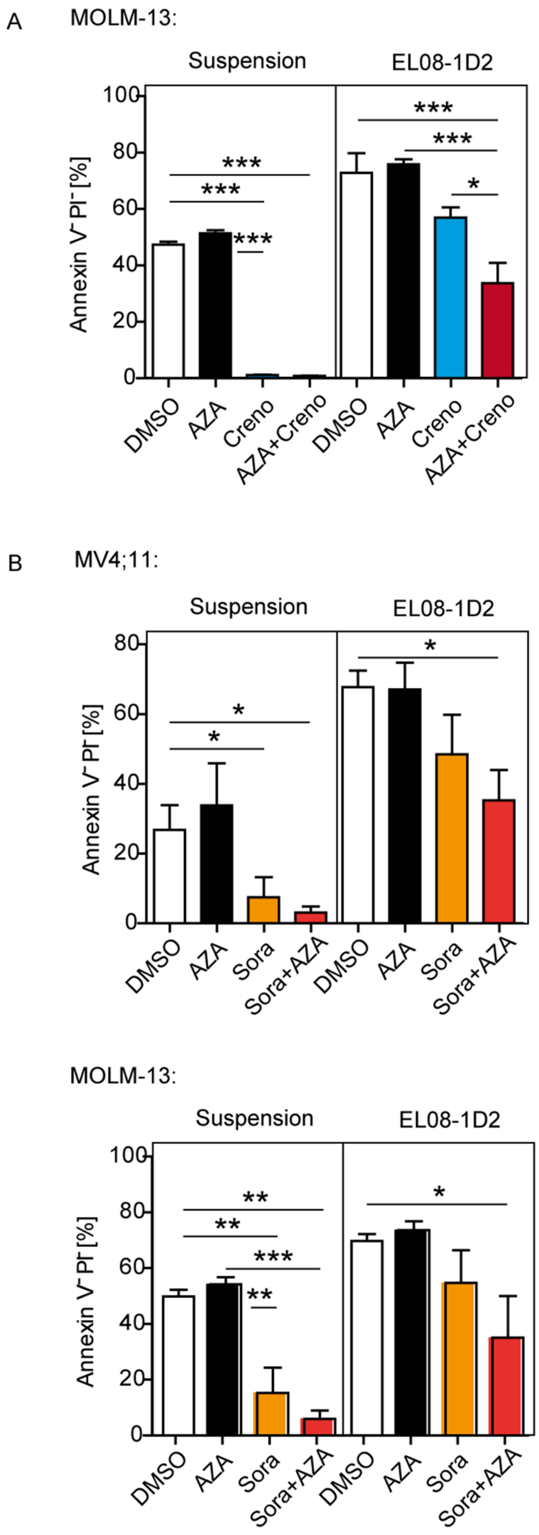

Supplementary Figure 4: Addition of AZA to TKI overcomes stromal resistance of *FLT3*-ITD AML cells. MV4-11 or MOLM-13 cells were cultured in suspension or on stromal EL08-1D2 layers. Cultures were treated with DMSO, AZA (10µM), TKI (100 nM creno or sora) or combination of AZA and creno for 8 days. Induction of apoptosis in AML cell lines was assessed using Annexin V/PI flow cytometry. Results are shown as mean of three independent experiments ± SEM. Crenolanib efficacy in MOLM-13 cells **(A)**. Sorafenib efficacy in MV4-11 and MOLM-13 cells **(B)**.

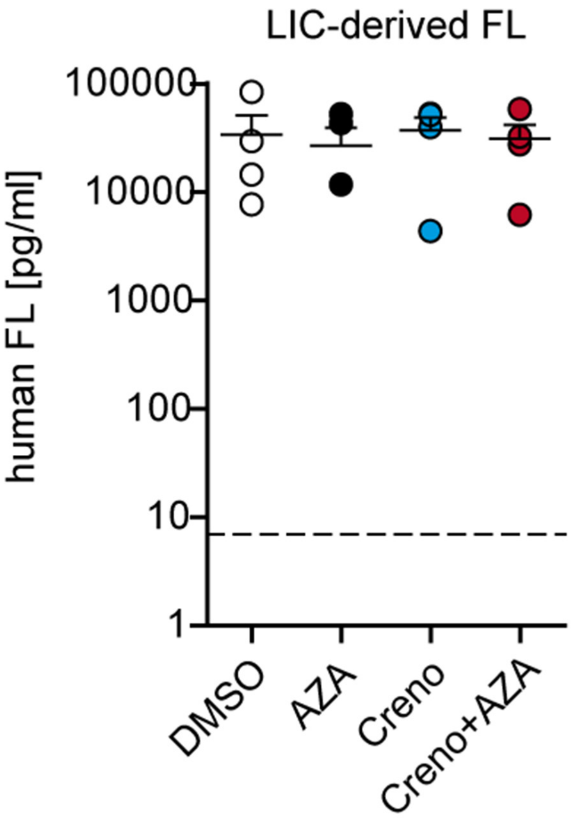

Supplementary Figure 5: FL does not account for stromal resistance of *FLT3*-ITD LIC. CD34^{+}-enriched *FLT3*-ITD AML BM cells were cultured on murine stromal EL08-1D2 layers and treated with DMSO, AZA (10 µM), creno (100 nM) or the combination thereof. After 24 hours, supernatants were collected and FL levels were measured using human-specific ELISA kit (R&D Systems) according to the manufacturer's instructions. Optical density was determined using the ELx800 Universal Microplate reader (BIO TEK Instruments, I.) and the Microplate Manager 5.2. software. Results are shown as mean of n=4 ± SEM.

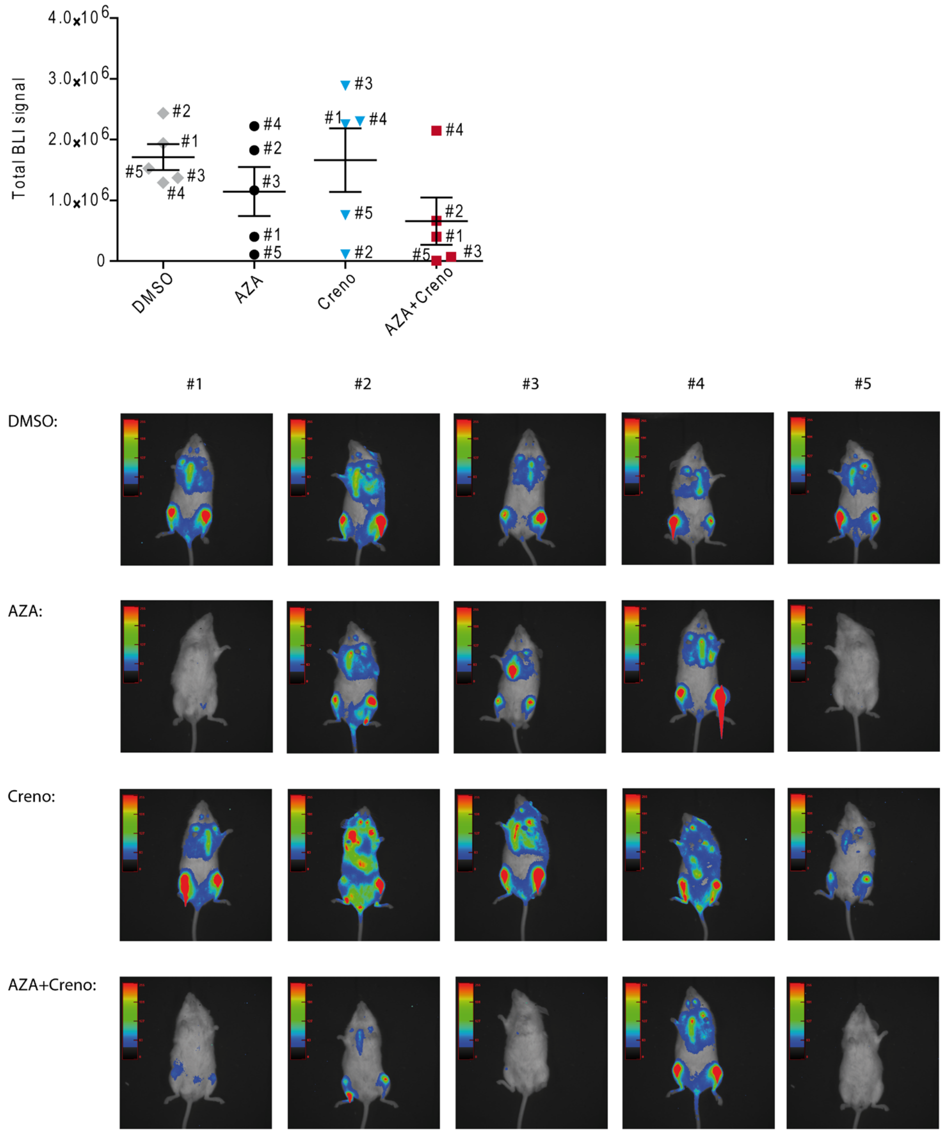

Supplementary Figure 6: AZA + creno reduces the *in vivo* engraftment capacity of *FLT3*-ITD/*NPM1*mut/*TET2*mut LIC in NSG mice. *FLT3*-ITD/*NPM1*mut/*TET2*mut transgenic-PDX cells were cultured *in vitro* on EL08-1D2 stroma and treated with DMSO, AZA, creno or the combination thereof for 4 days. T-PDX cells were harvested and 2 x 10^5 viable cells were injected IV into NSG mice (n=5 per cohort). Bright field grayscale images of each mouse were taken and bioluminescent signals were displayed in pseudocolors and projected on the grayscale images. Mean signal intensities mean values of each cohort (upper panel) and representative images of individual mice and are shown (lower panel).

Supplementary Data: Linear Regression Analyses for Figure 2C and Figure 3B

Supplementary File 1

Supplementary Data: Differential gene expression analysis of EL08-1D2 samples in co-culture compared to DMSO (Comparison 1)

Supplementary File 2

Supplementary Data: Differential gene expression analysis of PDX samples in co-culture compared to DMSO (Comparison 1)

Supplementary File 3

Supplementary Data: Differential gene expression analysis of EL08-1D2 samples in co-culture compared to monoculture (Comparison 2)

Supplementary File 4

Supplementary Data: Differential gene expression analysis of PDX samples in co-culture compared to monoculture (Comparison 2)

Supplementary File 5

Supplementary Data: Top 100 differentially expressed genes in EL08-1D2 cells. Gene ontology and KEGG pathway analysis

Supplementary File 6

Supplementary Data: Top 100 differentially expressed genes in PDX cells. Gene ontology and KEGG pathway analysis

Supplementary File 7
